# Precision Cancer Therapy Enabled Anti-Epidermal Growth Factor Receptor-Conjugated Manganese Core Phthalocyanine Bismuth Nanocomposite for Dual Imaging-Guided Breast Cancer Treatment

**DOI:** 10.34133/bmr.0092

**Published:** 2024-11-07

**Authors:** Sudip Mondal, Sumin Park, Van Tu Nguyen, Vu Hoang Minh Doan, Jaeyeop Choi, Cao Duong Ly, Duc Tri Phan, Thi Thuy Truong, Tan Hung Vo, Dinh Tuan Nguyen, Umapada Pal, Byeongil Lee, Junghwan Oh

**Affiliations:** ^1^ Digital Healthcare Research Center, Pukyong National University,; Busan 48513, Republic of Korea.; ^2^ Industry 4.0 Convergence Bionics Engineering, Department of Biomedical Engineering, Pukyong National University, Busan 48513, Republic of Korea.; ^3^ Smart Gym-Based Translational Research Center for Active Senior’s Healthcare, Pukyong National University, Busan 48513, Republic of Korea.; ^4^ Institute of Physics, Autonomous University of Puebla, Puebla, Pue. 72570, Mexico.; ^5^ Department of Smart Healthcare, Pukyong National University, Busan 48513, Republic of Korea.; ^6^ Ohlabs Corp., Busan 48513, Republic of Korea.

## Abstract

Cancer remains a formidable global health challenge, demanding the exploration of innovative treatment modalities with minimized side effects. One promising avenue involves the synergistic integration of targeted photothermal/photodynamic therapy (PTT/PDT), utilizing specially designed functional nanomaterials for precise cancer diagnosis and treatment. This study introduces a composite biomaterial, anti-epidermal growth factor receptor-conjugated manganese core phthalocyanine bismuth (anti-EGFR-MPB), synthesized for precise cancer imaging and treatment. The biomaterial, synthesized via a solvothermal process, effectively treats and images breast cancer in mouse models. Its biomimetic design targets cancer cells precisely, with dual imaging for real-time monitoring. The biomimetic design of the composite enables precise targeting of cancer cells, whereas the dual imaging allows for real-time visualization and monitoring of the treatment. In vivo examinations confirm substantial damage to tumor tissues with no recurrence following 808-nm laser irradiation. The composite shows strong fluorescence/photoacoustic imaging (PAI) contrast, aiding malignancy detection. Biological assays and histological analyses confirmed the efficacy of the nanocomposite in inducing apoptosis in cancer cells. The integrated targeted dual image-guided phototherapy offered by this composite substantially enhances the precision and efficacy of cancer therapy, achieving an impressive photothermal efficiency of ~33.8%. Our findings demonstrate the utility of the anti-EGFR-MPB nanocomposite for both in vitro and in vivo photoacoustic image-guided PTT and PDT. The optimal treatment strategy for triple-negative breast cancer is found to be the use of 250 μg/ml of nanocomposite irradiated with 1.0 W/cm^2^ 808-nm laser for 7 min.

## Introduction

The continuous evolution of innovative biomaterials and technologies presents promising avenues for addressing the unmet needs in women’s health, particularly in the realm of breast cancer. As one of the most prevalent and formidable diseases affecting women worldwide, breast cancer necessitates inventive strategies to enhance diagnosis, treatment, and patient outcomes [1,2]. Recent advances in drug delivery approaches are revolutionizing breast cancer management by offering tailored applications to address individual patient needs. From targeted therapies delivering medications precisely to tumor sites to localized drug release systems minimizing systemic side effects, these innovative approaches are reshaping the breast cancer treatment landscape [3,4]. Moreover, the integration of cutting-edge technologies such as nanomedicine, advanced imaging, and photothermal/photodynamic therapies (PTT/PDT) holds immense promise for improving drug delivery efficacy and personalizing treatment strategies [5]. Managing cancer clinically with noninvasive and effective treatments that incur minimal or no side effects necessitates the exploration of novel techniques. PTT emerges as a compelling alternative for cancer treatment due to its precision, minimal side effects, and enhanced efficacy [6].

Future strategies for disease management, including cancer treatment, are poised to heavily rely on advanced, tailored, and target-specific nanomedicine [7]. The unique attributes of nanomaterials enable early and precise disease detection with minimal patient disruption [8]. These nanomedicines can be synergistically combined with pharmaceuticals and contrast agents for simultaneous imaging and therapeutic purposes [9], facilitating treatment site visualization, drug accumulation assessment, and treatment progress monitoring [10].

Conventional supplementary drugs often inadvertently harm noncancerous cells [11], falling short of medical standards and posing challenges in translating them into real-time clinical applications. Recent advancements in radical materials show promise for biomedical applications such as imaging, sensing, and photo-triggered therapies. Enhancing the range of radical materials to include those with near-infrared (NIR) emission/absorption is preferred for reduced interference, deeper tissue penetration, and decreased harm. Combining innovative therapies like chemodynamic therapy (CDT), which leverages Fenton or Fenton-like reactions within the tumor microenvironment (TME) to generate harmful reactive oxygen species (ROS), with photo-induced therapies like PDT and PTT can synergistically enhance treatment efficacy and overcome multidrug resistance. CDT is minimally invasive, is highly tumor specific, and responds to overexpressed hydrogen peroxide (H_2_O_2_) and glutathione (GSH) in the TME. On the other hand, photoacoustic and fluorescence imaging are valuable modalities in biomedical research. PAI allows for deep tissue imaging with high spatial resolution, while fluorescence imaging offers real-time visualization of specific molecular targets, providing comprehensive insights into various diseases [12].

PTT is a promising option for cancer treatment due to its precise local destruction, noninvasive approach, and safety [13]. Efficient cancer treatment relies on using NIR lasers to irradiate nanoparticles (NPs) or nanocomposite [14,15]. Theranostic probes can be used for personalized therapy by combining photothermal energy with the controlled optical characteristics of nanomaterials [16]. The photothermal effect, which enables noble metal NPs to convert light into heat, is a promising method for hyperthermia-mediated tumor therapy [17,18]. Mn^2+^ ions within the nanocomposite material facilitate the catalytic conversion of abundant H_2_O_2_ in the TME into oxygen (O_2_) [19–21]. This mechanism alleviates tumor hypoxia and enhances the effectiveness of PDT [22,23].

Recently, Wang et al. [24] introduced a novel photosensitizer, manganese (Mn) phthalocyanine, which exhibits significant NIR absorption capacity and is used as a photosensitizer in PDT. It absorbs light at specific wavelengths and generates ROS that can damage or even kill cancer cells. Phthalocyanine’s ability to absorb light at specific wavelengths and produce ROS makes it an ideal candidate for PDT, allowing for the targeted destruction of cancer cells with minimal harm to healthy tissues [25,26].

Zhao et al. [27] developed nanostructured phthalocyanine assemblies and utilized them in PDT to treat tumors through PDT. As PDT is a cancer treatment method that uses light and a light-sensitive drug to destroy cancer cells [28], utilization of nanostructured phthalocyanine assemblies for this purpose not only enhanced penetration of the exciting light into the deep-lying tissues but also overcame tumor hypoxia through the generation of ROS.

On the other hand, bismuth (Bi)-based nanomaterials have shown remarkable capabilities in absorbing NIR light and consistently display significantly higher photothermal conversion efficiencies when compared to traditional photothermal agents such as Au-, Ag-, Cu-, or Pt-based NPs. Moreover, Bi-based nanomaterials offer cost-effectiveness and pose fewer hazards than metal-based photothermal agents. Their effectiveness as contrast agents in x-ray computed tomography (CT) imaging has garnered recognition, thereby enhancing visibility and improving image contrast for precise visualization of anatomical structures and pathological abnormalities during x-ray CT examinations. Furthermore, there have been reports on the utilization of Mn core phthalocyanine bismuth (MPB) composite for PTT in cancer treatment. Wang et al. synthesized phthalocyanine Mn nanocomposite tailored for biomedical imaging applications. The authors highlighted the stability and biocompatibility of these nanocomposites, rendering them suitable as contrast agents for in vivo imaging. However, limitations persist, including inadequate targeting specificity and limited circulation time within the body, leading to a reduction in overall efficacy. While intravenous injection remains the most effective clinical administration method, it often results in nonspecific binding, potentially causing collateral harm to noncancerous cells. Addressing these challenges is crucial to adapting these formulations for real-time clinical applications and meeting expected medical standards.

**Fig. 1.**
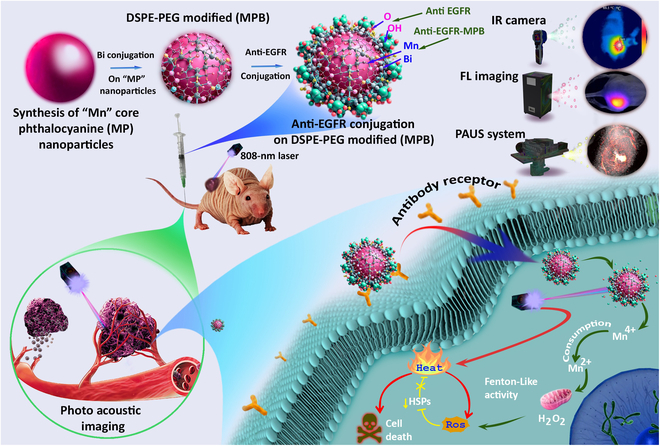
Schematic representation of anti-EGFR-MPB nanocomposite with molecular targeted therapeutic effect on MDA-MB-231 breast cancer cell line.

This study aims to synthesize an anti-epidermal growth factor receptor (EGFR)-conjugated MPB (anti-EGFR-MPB) composite for cancer treatment, integrating targeted dual image-guided phototherapy and precision medicine. This approach mimics natural biological interactions to combat cancer effectively. The anti-EGFR conjugation enhances precision by allowing specific recognition and binding to cancer cells, while the composite incorporates Mn core phthalocyanine for precise tumor imaging and real-time treatment monitoring. This combination of targeted imaging and localized phototherapy represents a breakthrough in cancer treatment, offering prospects for precision medicine by tailoring treatment strategies based on individual tumor characteristics. The ability of the composite to target cancer cells provides real-time imaging. It offers personalized treatment plans, with optimum therapeutic outcomes and minimum side effects. The ultrasound (US) photoacoustic dual image-guided PTT in a breast cancer mouse model with the newly synthesized anti-EGFR-MPB nanocomposite (Fig. 1) could be a promising therapeutic approach with enhanced targeted efficiency.

## Materials and Methods

### Chemicals and reagents

The chemicals and reagents utilized in this work are described in the Supplementary Materials.

### Synthesis of 1,8,15,22-tera-(3-(4-ethylcarboxyl-phenoxy)) phthalocyanine manganese (II)

The procedure adopted for the synthesis of 1,8,15,22-tera-(3-(4-ethylcarboxyl-phenoxy)) phthalocyanine manganese (II) (MP) has been described in the Supplementary Materials.

### Synthesis of MPB nanocomposite and their PEG functionalization

The procedures adopted for the synthesis of MPB nanocomposite and their polyethylene glycol (PEG) functionalization have been described in the Supplementary Materials.

### Conjugation of MPB nanocomposite with anti-EGFR

Anti-EGFR was conjugated to Bi phthalocyanine Mn nanocomposite with some modifications of the previously published reports [29]. By using PEG-conjugated esters to react with the primary amines on the anti-EGFR antibody, it is possible to create an anti-EGFR-MPB nanocomposite. The synthesized final product was further incubated at room temperature for 2 to 3 h to allow the antibodies to bind to the nanocomposite. The anti-EGFR-MPB particles were separated by centrifugation to remove unbound antibodies. The procedure was done multiple times until reaching the intended degree of conjugation.

### In vitro cytotoxicity study of MPB nanocomposite

For the in vitro cytotoxicity assessment of the MPB nanocomposite, we conducted an MTT [3-(4,5-dimethylthiazol-2-yl)-2,5-diphenyltetrazolium bromide] assay. MDA-MB-231 breast cancer cells were cultured in a 96-well plate and incubated for 24 h. The concentrations of the MPB nanocomposite were set at 0, 50, 100, 150, 200, 250, and 300 μg/ml. Additionally, we evaluated the in vitro cytotoxicity of the MPB nanocomposite using acridine orange/propidium iodide (AO/PI) and Hoechst 33342/PI labeling techniques. In these methods, the nuclei of dying cells were stained red with PI, while live cells were marked with AO (green) and Hoechst 33342 appeared blue. Following a 24-h incubation period, MDA-MB-231 cells formed a monolayer in a 6-well plate. Subsequently, NIR irradiation treatment was performed on the cells at various concentrations and durations. After the irradiation treatment, the cells were stained with an AO/PI solution for 10 min. Finally, cellular images were captured using confocal laser scanning microscopy (CLSM).

### Flow cytometry analysis of cell death

The experimental procedure commenced by immersing the cells in an MPB nanocomposite solution for 6 h. Following this, the cells underwent laser irradiation at a specific intensity for 7 min. After the laser treatment, the cells were further incubated for 6 h under controlled temperature and environmental conditions. Subsequently, the cells were harvested, washed with a cold solution, and stained using the FITC-Annexin V Apoptosis Detection Kit according to the manufacturer’s instructions. To evaluate cell death, flow cytometry was conducted using the BD FACSVerse flow cytometer as per the manufacturer's guidelines. The results were analyzed using FlowJo software (Ashland, OR, USA). The flow cytometry experiment used the dedicated BD FACSVerse flow cytometer.

### In vitro cellular uptake studies

In 12-well plates, MDA-MB-231 cells were seeded with 1 × 10^5^ cells per well and allowed to incubate overnight. Following incubation, the cells were exposed to culture medium containing MPB at a concentration of 250 μg/ml. At specific time intervals, the cells were washed with phosphate-buffered saline (PBS) and then counted using a hemocytometer. To conclude the experiment, cell pellets were treated with 400 μl of HNO_3_, and the content of Bi/Mn was determined via inductively coupled plasma mass spectrometry (ICP-MS) analysis. The cellular uptake of MPB was further confirmed through transmission electron microscopy (TEM) and energy-dispersive x-ray spectroscopy [biological-transmission electron microscope (B-TEM) analysis].

### Animal model

A group of female BALB/c nude mice, weighing approximately 18.9 ± 0.8 g and aged between 6 and 7 weeks, was procured from Orient Bio Inc. in Seongnam, Republic of Korea. The details of animal model experiments are presented in Table S1.

### Histological study

After the 3-week treatment period, all the mice from each experimental group were euthanized. The study commenced on reaching the desired tumor size, observed 28 d after the experiment commenced. Tumor specimens, along with vital organs (heart, lungs, spleen, kidney, and liver), were collected for examination. To prepare and preserve the collected organs, they were first rinsed with saline solution and subsequently immersed in 10% formalin. After 24 h of fixation, the tissues were embedded in paraffin and sectioned to a thickness of 4 μm. These sections were subsequently stained with hematoxylin and eosin (H&E) and examined using an optical microscope for histopathological analysis.

### PAI and in vivo PTT

Recently, noninvasive biomedical imaging has emerged as a potential diagnostic method [30,31]. PAI stands out among other imaging techniques as it allows for the simultaneous identification and quantification of NP contrast agents within biological systems. In this study, we utilized an in vivo photoacoustic microscopy system (FPAM-v1, Ohlabs, Republic of Korea) for PAI, following the protocols outlined in previous publications. Detailed information on the methodology is provided in the Supplementary Materials.

### Fluorescence imaging

The synthesized MPB nanocomposite was excited using a 635-nm excitation wavelength, and the emission spectra were recorded at 703 nm [32]. Subsequently, to capture images, the emitted signals from the sample were filtered and captured by the imaging module. Additional details regarding this process are presented in the Supplementary Materials.

### Western blotting tests

Western blotting technique is a potent analytical method used for the detection and quantification of proteins in complex biological samples. The general protocol utilized for conducting Western blot tests is presented in the Supplementary Materials.

### Statistical analysis

All data were presented as mean values accompanied by their respective standard deviations. The statistical analysis was conducted through a one-way analysis of variance (ANOVA). Data processing was carried out using OriginPro 8.0 software developed by OriginLab Corp. (Northampton, MA, USA).

### Ethics approval and consent to participate

All animal handling procedures adhered to the guidelines set forth by Pukyong National University, Busan, South Korea, and were conducted in compliance with authorized institutional policies and procedures for animal care services and experimentation (permit number: PKNUIACUC-2022-16).

## Results

### X-ray powder diffraction

**Fig. 2.**
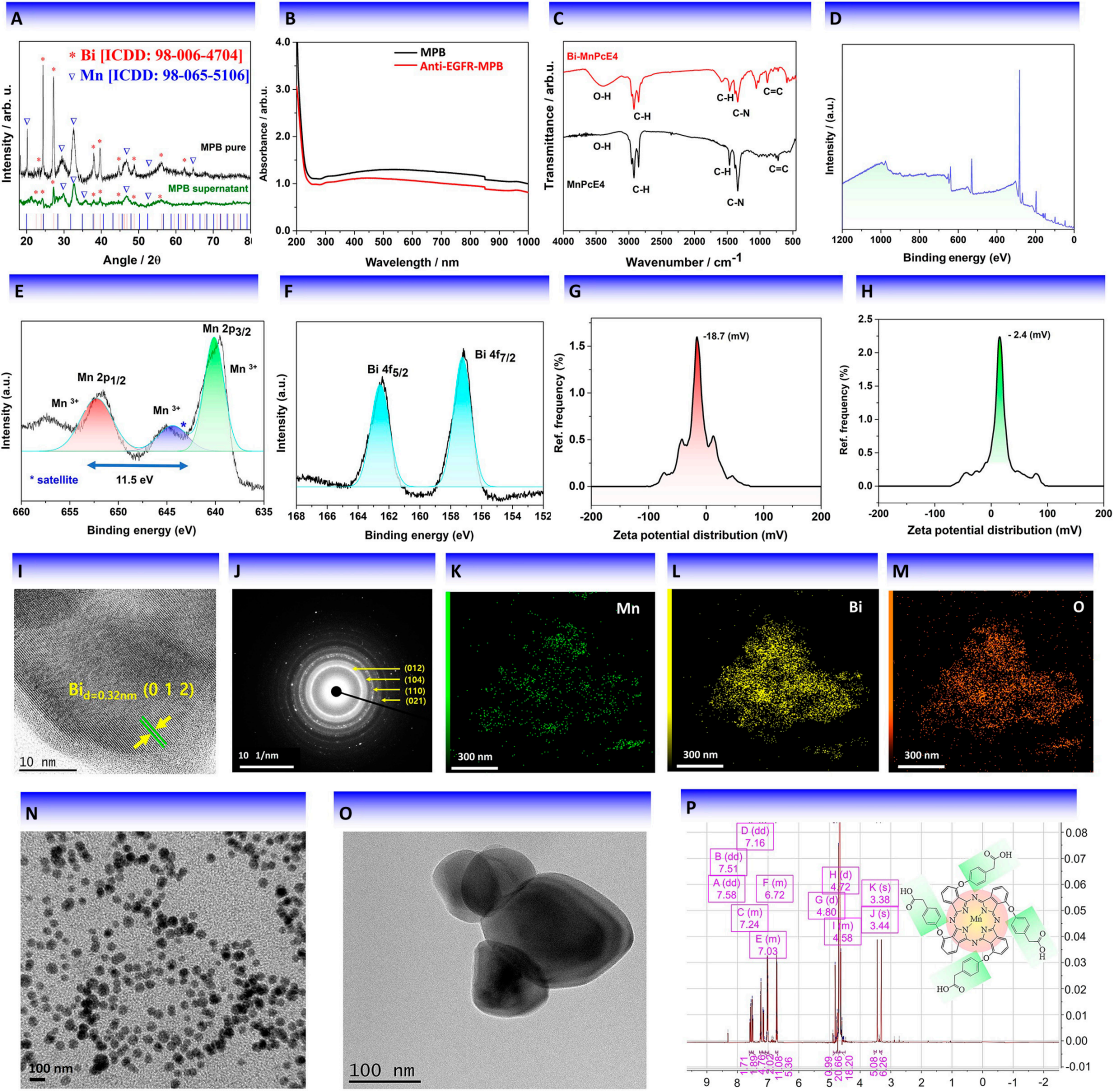
Characterization of anti-EGFR-MPB nanocomposite by (A) XRD, (B) UV-vis, and (C) FTIR spectra of MP and MPB. (D to F) Survey and core-level XPS spectra of MPB nanocomposite. (G) Zeta potential distribution for MPB nanocomposite. (H) Zeta potential distribution for anti-EGFR-MPB nanocomposite. (I) A typical HR-TEM image of the composite anti-EGFR-MPB particle, revealing its good crystallinity and Bi core. (J) SAED pattern [taken over high-contrast Bi region of the image presented in (I) of the typical anti-EGFR-MPB NP]. (K to M) EDS elemental mapping of anti-EGFR-MPB nanocomposite, revealing the distribution of Mn, Bi, and O elements. (N and O) Typical TEM images of the anti-EGFR-MPB nanocomposite. (P) ^1^H NMR spectrum of MP.

X-ray powder diffraction (XRD) study was conducted to analyze the crystalline structure of the synthesized MPB nanocomposite. XRD patterns of the pure MPB and the MPB sample collected from the supernatant (Fig. 2A) recorded in the 18° to 80° range of 2*θ* revealed sharp peaks at approximately 38.46°, 45.14°, 64.67°, and 77.83°, which correspond to the (1 1 1) lattice planes of Bi (ICDD card no. 98-006-4704) and (2 0 0), (2 2 0), and (3 1 1) lattice planes of Mn (ICDD card no. 98-065-5106) in the face-centered cubic (fcc) phase. The estimated interplanar spacing (*d*_cal_) values associated with these peaks are 2.334, 1.952, 1.429, and 1.221 Å, respectively.

### UV-visible spectroscopy and FTIR analysis

Visual observation revealed MPB to be a black color material. Ultraviolet-visible (UV-vis) spectroscopy analysis of the pristine (nonfunctionalized) and anti-EGFR functionalized MPB samples revealed a broad absorption band spanning from 350 to 1,150 nm (Fig. 2B), corresponding to the S_0_–S_1_ transition in MnPcE_4_ [33]. The successful formation of the MPB nanocomposite was validated using FTIR (Fourier transform infrared) spectroscopy. The FTIR spectra of the MP and MPB samples (Fig. 2C) revealed characteristic absorption peaks of methylene group (C–H bending) vibration at 1,465 cm^−1^ and the hydroxyl (-OH) stretching vibration at 3,510 cm^−1^. The peak at around 1,610 cm^−1^ indicates the C=C stretching vibration in the MPB sample. Notably, the absorption peaks corresponding to methylene (C–H) groups in the MPB nanocomposite were more pronounced, possibly due to the presence of DSPE-PEG3000 [1,2-distearoyl-sn-glycero-3-phosphoethanolamine-*N*-maleimide(polyethylene glycol)3000] introduced during the synthesis process.

### X-ray photoelectron spectroscopy

An x-ray photoelectron spectroscopy (XPS) study was conducted to analyze the surface composition of the pristine MPB nanocomposite. The XPS survey spectrum of the pristine MPB sample (Fig. 2D) revealed 2 prominent peaks at binding energies of 162 and 639 eV, corresponding to Bi 4f and Mn 2p emissions, respectively. The spectrum revealed emissions corresponding to O1s and C1s orbitals (Fig. 2D) along with the emissions from the Bi 3d and Mn 3d orbitals. It should be noticed that Mn in the composite remained in the Mn^3+^ state, while Bi remained in the metallic (Bi^0^) state. The core-level spectra of both the metals displayed spin-orbit splitting of the 3d orbital (Fig. 2E and F).

### Dispersion stability and zeta potential study

To assess the interaction of the nanocomposite with water, it was dispersed in PBS, Dulbecco’s modified Eagle’s medium (DMEM), and fetal bovine serum (FBS). Remarkably, after 48 h, the anti-EGFR-MPB nanocomposite demonstrated excellent stability across various concentrations (Fig. S1A and B), indicating its high stability in the biological environment. Furthermore, the stability of the anti-EGFR-MPB nanocomposite dispersed in PBS was evaluated using dynamic light scattering (DLS) at different incubation times.

The zeta potential and distribution of hydrodynamic particle sizes in the prepared MPB nanocomposite were also examined. The MPB nanocomposite exhibited a zeta potential of approximately −18.7 mV (Fig. 2G and H) and a hydrodynamic size of approximately 113 ± 12.7 nm (Fig. S1). This observation might be attributed to the presence of functional groups or other chemical species that ionize in the suspension medium, such as carboxylic acid groups. On the other hand, the anti-EGFR-MPB nanocomposite displayed a lower negative zeta potential of around −2.4 mV (Fig. 2H). This reduction in zeta potential could be due to the modification of the particle surface with anti-EGFR antibodies, potentially masking some of the negatively charged groups and diminishing the overall negative charge of the particles. Additionally, the presence of antibodies might induce a steric hindrance effect, leading to a decrease in the effective surface charge. The stability of the synthesized MPB nanocomposite was further confirmed by incubating it in PBS, FBS, and DMEM solutions. Results depicted in Fig. S2 confirm the sustained stability of the MPB nanocomposite in the tested biological medium.

### Transmission electron microscopy

TEM imaging provides crucial insights into the nanostructure and composition of materials. High-resolution TEM (HR-TEM) analysis (Fig. 2I) revealed an interplanar distance of 0.32 nm, corresponding to the Bi (0 1 2) planes. Bright spots observed on concentric diffraction rings in the selected-area electron diffraction (SAED) pattern (Fig. 2J) denote the lattice planes (0 1 2), (1 0 4), (1 1 0), and (0 2 1) of Bi metal. The energy-dispersive spectroscopy (EDS) analysis of MPB was employed to confirm the ratio of Bi/Mn in the produced nanocomposite (Fig. S3B). The peaks observed at approximately 8 and 9 keV correspond to the emission of Cu Kα and Cu Kβ, respectively, of the copper grid in the TEM. As can be noticed in Fig. 2N and O, the produced composite NPs are smaller than 150 nm in size and of quasi-spherical shape. However, the MPB particles experienced a shape distortion following photothermal processing (Fig. S3). Upon utilization in PTT, the composite MPB NPs displayed a distinct size distribution, with an average of 97.4 ± 28 nm. The synthesized nanostructures were well dispersed in PBS (Fig. 2N). Mn phthalocyanine is a complex organic molecule, commonly studied using nuclear magnetic resonance (NMR) spectroscopy [34]. The molecular structure of Mn phthalocyanine consists of a central Mn ion surrounded by a macrocyclic ring of nitrogen and multiple aromatic rings. NMR spectra of Mn phthalocyanine can provide detailed information about the molecular structure of this complex molecule. In the ^1^H NMR spectrum of Mn phthalocyanine, signals from aromatic protons appear as multiple peaks associated with several aromatic rings in the molecule. These peaks experience a chemical shift relative to the solvent in which the sample is dissolved, and the relative intensity of the peaks depends on the number of protons in each aromatic ring and their environment (Fig. 2P).

### Photothermal performance of MPB

**Fig. 3.**
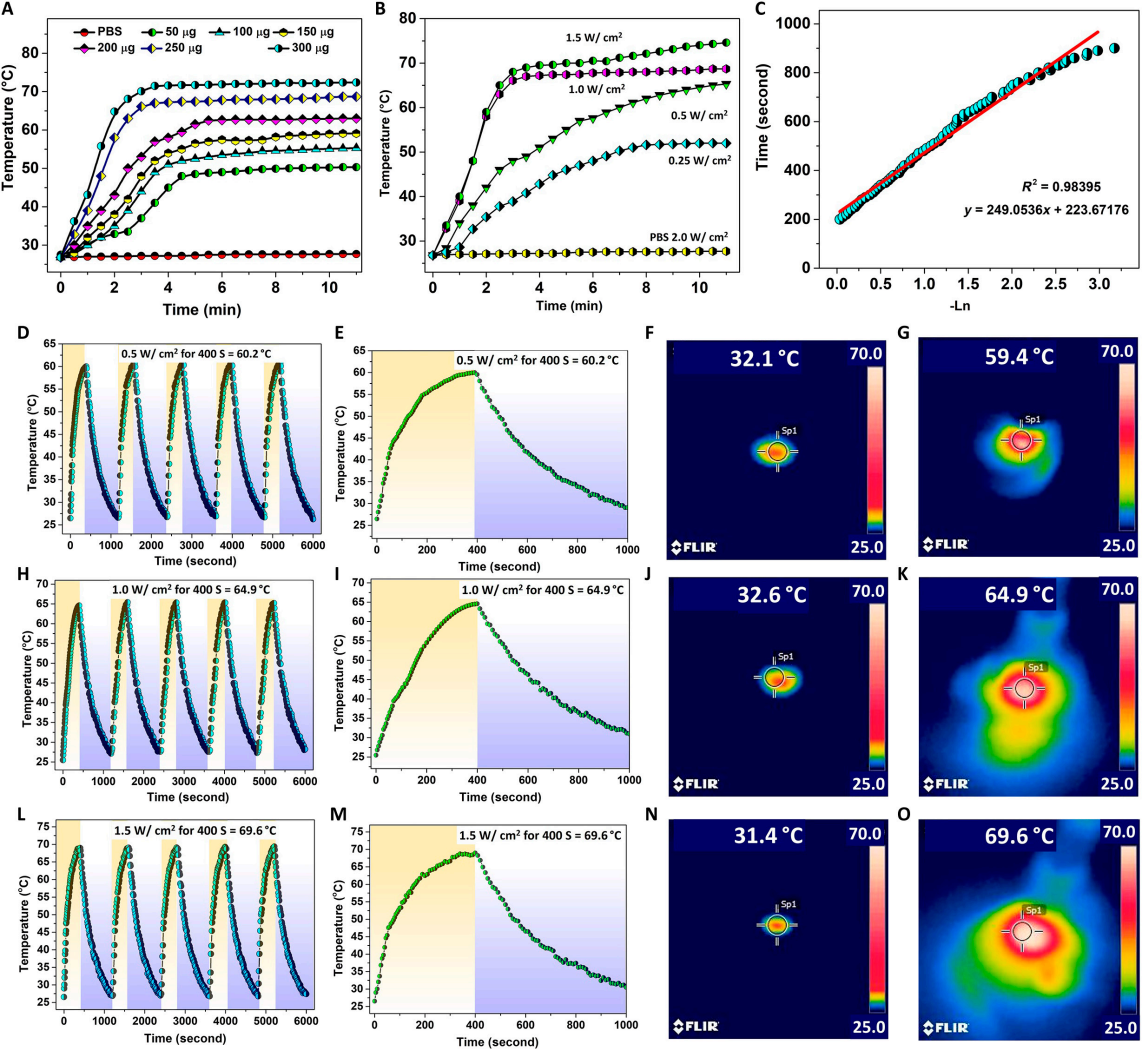
Photothermal performance of anti-EGFR-MPB nanocomposite. (A) Temperature evolution in anti-EGFR-MPB nanocomposite solutions of different concentrations on exposure to the 808-nm laser for 11 min at an optical power density of 1.0 W/cm^2^. (B) Thermal graph displaying the nanocomposite solution at a concentration of 250 μg/ml, exposed to an 808-nm laser source for 11 min across 4 distinct optical power densities (0.25, 0.5, 1.0, and 1.5 W/cm^2^). (C) Time constant curve fitting for heat transfer of MPB nanocomposite (250 μg/ml) (τs = 249.06 s) by applying linear time data versus −Ln(θ) from the cooling stage. (D) Temperature variation curve of the MPB nanocomposite solution of 250 μg/ml concentration under 808-nm laser irradiation for 5 cycles and (E) single-cycle temperature evolution curve at 0.5 W/cm^2^ laser power intensity. (F) Corresponding IR image before laser irradiation and (G) after laser irradiation. (H) Five-cycle temperature variation curve of the MPB nanocomposite at 1.0 W/cm^2^ laser power intensity. (I) Single-cycle temperature evolution curve at 1.0 W/cm^2^ laser power intensity. (J) Corresponding IR image before laser irradiation and (K) after laser irradiation. (L) Five-cycle temperature variation curve of the MPB nanocomposite at 1.5 W/cm^2^ laser power intensity. (M) Single-cycle temperature evolution curve at 1.5 W/cm^2^ laser power intensity. Corresponding IR images at (N) pre-laser irradiation and (O) post-laser irradiation.

To evaluate the photothermal performance of the prepared anti-EGFR-MPB nanocomposite, first, a 12-well cell culture plate was filled with nanocomposite solutions (in PBS) of various concentrations (50, 100, 150, 200, 250, and 300 μg/ml). Then, the solutions in the cell culture plate were irradiated with an 808-nm laser for 11 min at different optical densities. The temperature increase resulting from the photothermal effect was captured using an IR thermal camera. The temperature evolutions of the solutions over 11 min of laser irradiation at a power density of 1.0 W/cm^2^ are presented in Fig. 3A. Additionally, the temperature evolutions for a 250 μg/ml nanocomposite solution at different power densities are shown in Fig. 3B. The efficiency of converting light into heat for the MPB nanocomposite (~30.8%; Fig. 3C) was determined using the methods outlined in the additional materials provided, including Table S2 and Fig. S4 [35].

As shown in Fig. 3A, after 11 min of exposure at 1.0 W/cm^2^, the temperatures of solutions with concentrations of 50, 100, 150, 200, 250, and 300 μg/ml rose to 50.3, 55.3, 59.1, 63.0, 68.7, and 72.4 °C, respectively. In contrast, the final temperature of the PBS solution alone was only 27.7 °C (Fig. 3A). As demonstrated in Fig. 3B, the temperature rises in the 250 μg/ml MPB composite solution after 11 min of laser exposure at optical power densities of 0.25, 0.5, 1.0, and 1.5 W/cm^2^ reached up to 52.1, 65.3, 68.7, and 74.6 °C, respectively. For the PBS solution alone, the final temperature after 11 min of exposure at a laser power density of 2.0 W/cm^2^ was 28.2 °C [36]. When subjected to a 1.5 W/cm^2^ 808-nm laser, IR thermal imaging detected temperatures of up to 70.0 °C in the 250 μg/ml MPB composite solution (Fig. 3C). Throughout 5 on/off cycles, the temperature behavior of the 250 μg/ml MPB composite solution demonstrated consistent and stable performance (Fig. 3D, H, and L). Following laser irradiation, field-emission TEM and UV-vis spectroscopy were utilized to characterize the MPB nanocomposite further.

### Fluorescent staining and cell viability study

**Fig. 4.**
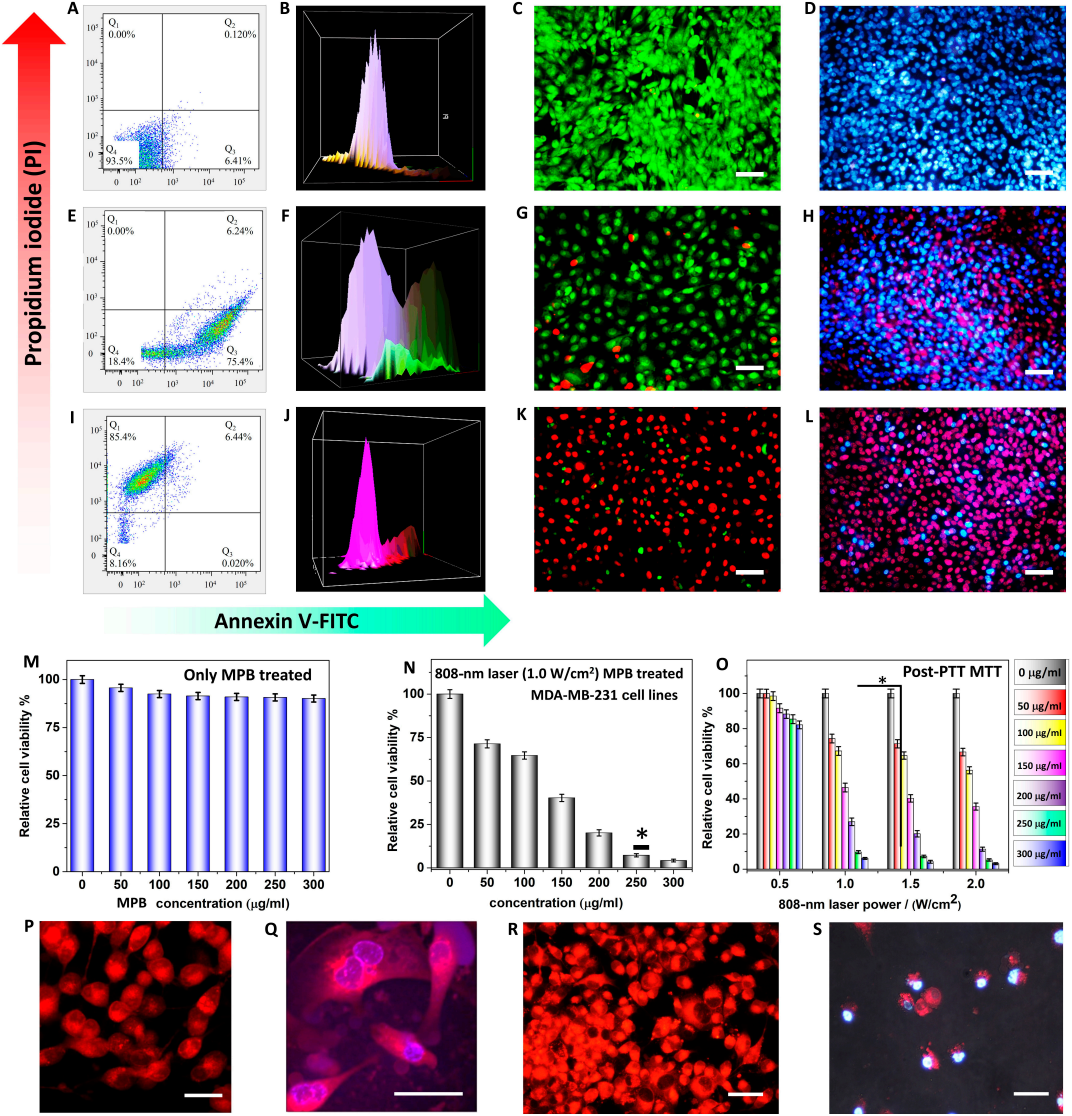
Analysis via flow cytometry to study apoptosis in MDA-MB-231 cells: (A and B) control (without nanocomposite or laser irradiation), (E and F) PDT induced by anti-EGFR-MPB nanocomposite with mild laser irradiation, (I and J) PTT study using the PI/Annexin V staining, and (C, G, and K) AO/PI study. (D, H, and L) Hoechst 33342/PI fluorescent staining using PI for live and dead cells was conducted on the MDA-MB-231 breast cancer cell line using various conditions. Scale bars, 50 μm (C, D, G, H, K, and L). Cell viability study of breast cancer cell lines: (M) following a 24-h incubation with varied concentrations of MPB nanocomposite, (N) after incubation in MPB nanocomposite solution and exposition to 808-nm laser (1.0 W/cm^2^ power density) for 11 min, and (O) after incubation in anti-EGFR-MPB nanocomposite solution and 11-min exposition to the laser at different optical power densities (0.25, 0.5, 1.0, and 1.5 W/cm^2^). Data are expressed as mean ± SD (*n* = 5, *P < 0.05 versus control). Post-PTT/PDT CLSM study with different fluorescent staining. (P to S) MitoTracker Red CMXRos/Hoechst 33342 study of MDA-MB-231 cell lines. Scale bars, 10 μm (P, R, and S) and 5 μm (Q).

In the fluorescent staining and cell viability study, colloidal solutions of MPB nanocomposite were prepared at a concentration of 250 μg/ml and exposed to MDA-MB-231 cells. For the comparison, MDA-MB-231 cells were also tested without exposure to the nanocomposite or the laser (Fig. 4A to D) as a control. Figure 4C and D shows AO/PI and Hoechst 33342/PI staining for the control group, which is discussed in more detail in the following section. Flow cytometric analysis was performed on MPB-treated cells, using MDA-MB-231 cells, both with and without exposure to a laser with a power density of 1.0 W/cm^2^ (Fig. 4A, B, E, F, I, and J). Figure 4G and H depicts AO/PI and Hoechst 33342/PI staining after PDT study discussed later in this section. The fluorescence-activated cell sorting study results revealed that approximately 93.5% of the cells in the control group were healthy, with around 6.41% in the early stages of apoptosis and approximately 0.120% in the late stages, while no cells were found in the necrosis stage. Mild laser exposure (PDT study) led to around 18.4% of MPB-treated cells remaining healthy, with about 75.4% in the early stages of apoptosis and approximately 6.24% in the late stages. The treated cells did not exhibit signs of necrosis.

However, exposure of MPB-treated cells to laser irradiation resulted in significant changes in cell survival. Only about 8.16% of the cells were detected as healthy, with around 0.020% in the early stages of apoptosis and about 6.44% in the late stages. Additionally, the photothermal effect induced necrosis in approximately 85.4% of the cells. PTT involves the use of light-absorbing agents, such as NPs or photosensitizers, which convert light energy into heat, leading to rapid and intense thermal ablation, direct cellular damage, disruption of cell membranes, and subsequent necrotic cell death, as well as triggering an inflammatory response.

PDT utilizes photosensitizers that, upon exposure to light, initiate the production of ROS such as singlet oxygen within cells. The oxygen (O_2_) generated through the catalytic decomposition of H_2_O_2_ can be harnessed in PDT and converted into singlet oxygen (^1^O_2_) species (Fig. S5). These ROS induce oxidative stress, leading to cellular damage and the activation of apoptotic pathways. Apoptosis is a meticulously controlled process of programmed cell death marked by distinct morphological and biochemical alterations, such as cellular shrinkage, nuclear fragmentation, and membrane blebbing (Fig. 4E). The generated heat in PTT results in a rapid and thermal ablation, causing direct cellular damage, disruption of cell membranes, and subsequent necrotic cell death. The release of intracellular contents due to disrupted membranes can trigger an inflammatory response (Fig. 4I).

In the AO/PI fluorescence staining study, most cells emitted green fluorescent light when observed under a fluorescence microscope, indicating their healthy, viable state. Similarly, Hoechst 33342 staining produced blue fluorescence in live cells. In this study, 2 different sets of counterstains were used: (a) AO/PI and (b) Hoechst 33342/PI. Red fluorescence, present only in cells treated with MPB, signified the presence of dead cells (Fig. 4G, H, K, and L). The uptake of the MPB nanocomposite within the cells was assessed through biological TEM analysis, presented in Fig. S6A and B. The adverse effects of high MPB concentrations in the MTT assay were observed, with dead cells emitting red fluorescence during fluorescence excitation. MDA-MB-231 cells treated with MPB exhibited a significant number of living cells emitting green light, as measured by AO fluorescence (Fig. 4C). The results of the MTT assay for treated MDA-MB-231 cells were consistent with these findings. When exposed to a laser with a wavelength of 808 nm and a power density of 1.0 W/cm^2^ at a concentration of 250 μg/l, the otherwise nontoxic MPB nanocomposite became toxic, as observed in Fig. 4K. The cells were exposed to laser light before being cultured with AO/PI fluorescent dyes. More than 97% of the cells in the study were found to be dead, with red fluorescence visible in all of the dead cells across the microscopic area.

### MTT assay for in vitro cytotoxicity study

In the MTT assay for in vitro cytotoxicity study, the MDA-MB-231 cell line was exposed to varying doses of the MPB nanocomposite for 24 h. The results revealed no detrimental effects of the nanocomposite on the cells (Fig. 4M to O). At the lowest dose (50 μg/ml) of the MPB nanocomposite, over 95% of the cells remained viable. At the highest concentration tested (300 μg/ml), only 10% of cell death was observed, with 87% remaining viable. Additionally, cells treated with the MPB nanocomposite at the same doses for 72 h exhibited enhanced viability, reaching approximately 86% at 100 μg/ml. Minimal toxicity was observed at a concentration of 150 μg/ml, with overall cell viability at approximately 95%. Even at 250 μg/ml, 89% of the cells remained viable (Fig. 4M)*.* A study was conducted to determine the impact of the nanocomposite on MDA-MB-231 cells exposed to photothermal effects (Fig. 4N). The results revealed more pronounced cytotoxic effects of the nanocomposite when exposed to laser light compared to the control. The level of toxicity of the nanocomposite increased when exposed to a laser with a power density of 1.0 W/cm^2^, resulting in a decrease in cell viability to approximately 70% for a low concentration of 50 μg/ml and as low as 4% for a high concentration of 250 μg/ml. Similar lethal effects were observed when cells were exposed to laser-exposed MPB nanocomposite (Fig. 4N and O). The nontoxic MPB nanocomposite generated sufficient heat when exposed to a laser, effectively destroying the cancer cells.

The anti-EGFR-MPB nanocomposite-treated cells were further examined using CLSM (Fig. 4P and R). Figure 4Q and S displays the CMXRos/Hoechst 33342 stain for the control group. In these images, the blue color indicates healthy cells, while CMXRos detects the changes in mitochondrial membrane potential.

### In vitro PDT

In vitro PDT harnesses ROS produced by photosensitizers, light, and oxygen to induce cancer cell death. Yet, the TME can pose challenges due to hypoxia (low oxygen level). Previous studies on Mn^2+^ ions revealed their ability to transform into Mn(OH)_2_ and MnO_2_ when exposed to H_2_O_2_ in a neutral environment. The unstable Mn(OH)_2_ can react further with H_2_O_2_ to generate MnO_2_. The formation of MnO_2_ significantly enhances its catalytic capacity to decompose H_2_O_2_, simultaneously producing oxygen and reducing MnO_2_ back to Mn^2+^. The following reactions elucidate the entire process:

$${\text{Mn}}^{2+}+2\;H_{2}O_{2}⇄\text{Mn}{\left(\text{OH}\right)}_{2}+{\text{2H}}^{+}$$ (1)

$$\text{Mn}{\left(\text{OH}\right)}_{2}+H_{2}O_{2}\to {\text{MnO}}_{2}+{\text{2H}}_{2}O$$ (2)

$${\text{MnO}}_{2}+H_{2}O_{2}+{\text{2H}}^{+}\to {\text{Mn}}^{2+}+{\text{2H}}_{2}O+O_{2}↑$$ (3)

**Fig. 5.**
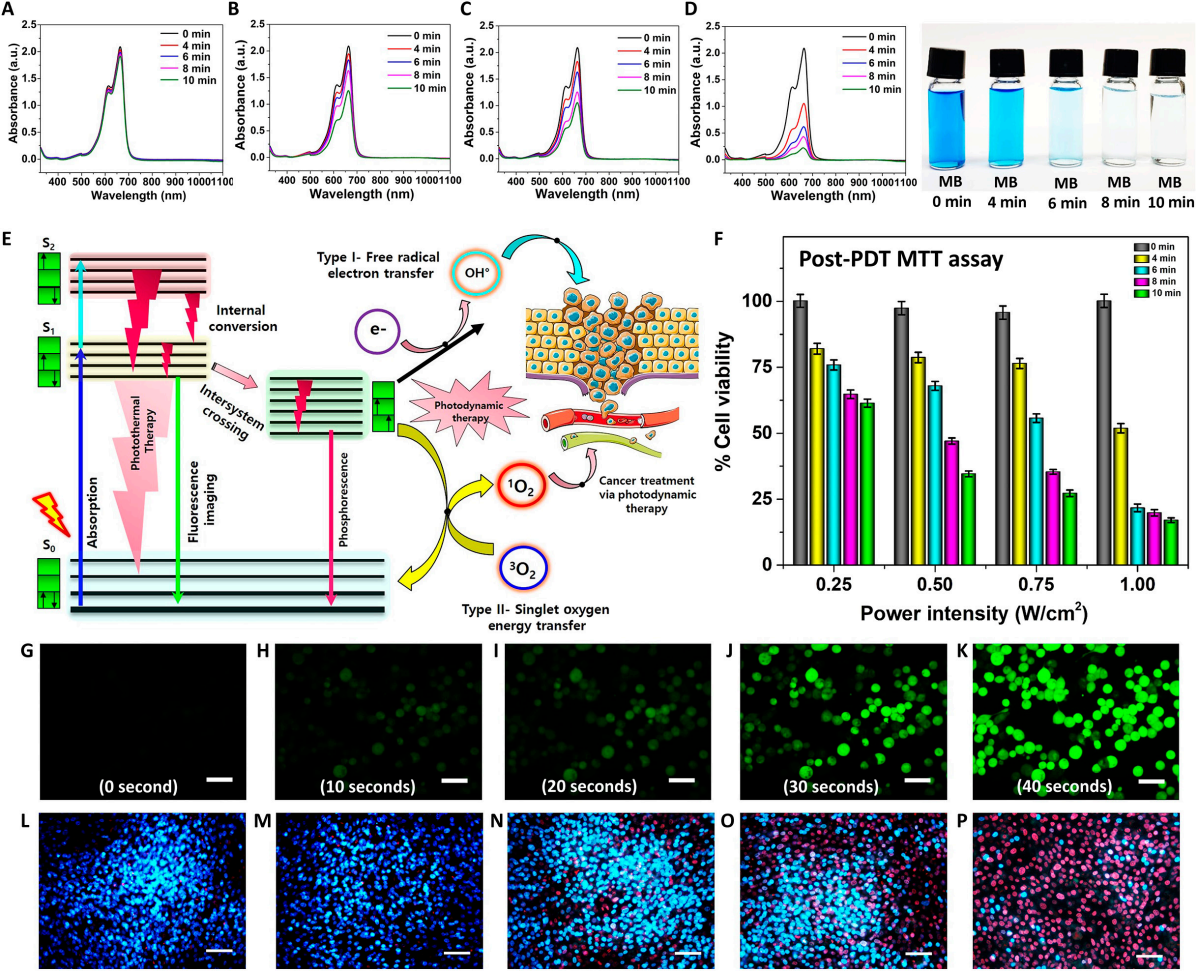
Photodynamic effect of anti-EGFR-MPB. UV-vis absorption spectra obtained from MB dye combined with various treatments for different irradiated times: (A) without laser irradiation, and 11-min laser irradiation (B) at 0.25 W/cm^2^, (C) 0.5 W/cm^2^, and (D) 1.0 W/cm^2^ power densities. The degradation of MB with 1.0 W/cm^2^ laser irradiation at different time intervals due to the generation of ROS. (E) Jablonski diagram, encompassing vibrational levels for absorption, nonradiative decay, and fluorescence in a schematic format. (F) In vitro post-photodynamic study MTT assay of anti-EGFR-MPB nanocomposite (250 μg/ml)-incubated MDA-MB-231 cells on 808-nm laser exposure at 1.0 W/cm^2^. (G to K) Detection of the production of singlet oxygen (^1^O_2_) resulting from the PDT effect by reacting with DCFH-DA, using the synthesized nanocomposite. Scale bars, 20 μm. (L to P) Fluorescent images of Hoechst 33342/PI-stained cells showing the viable cells (blue) and dead cells (red) after different treatments.

The oxygen (O_2_) generated through the catalytic decomposition of H_2_O_2_ can be harnessed in PDT and converted into singlet oxygen (^1^O_2_) species. To assess oxygen production, we employed 1,3-diphenylisobenzofuran (DPBF) as a probe. UV-vis absorption spectra were used to measure singlet oxygen (^1^O_2_) production. The UV absorbance of DPBF combined with a methylene blue (MB) solution was utilized as a reference to determine the efficacy of generating singlet oxygen (^1^O_2_) (Fig. S5). The PDT performance of the MPB nanocomposite under laser irradiation was enhanced by the addition of a small quantity of H_2_O_2_, as seen in Fig. 5A to C. To verify the PDT mechanism, MB dye was employed to confirm the catalytic properties of the synthesized nanocomposite. In the absence of laser irradiation, the MPB nanocomposite did not exhibit significant O_2_ generation or degradation of MB, as shown in Fig. 5D. However, a slight degradation of MB was observed, likely due to the adsorption associated with MPB nanocomposite. The Jablonski diagram presented in Fig. 5E illustrates the singlet oxygen generation potential of the nanocomposite through type I and II photoreaction mechanisms.

In vitro studies were conducted using 250 μg/ml MPB nanocomposite solutions on MDA-MB-231 cells. The cells were treated with the MPB nanocomposite solution and irradiated with an 808-nm laser for varying durations. The culture medium was supplemented with the MPB nanocomposite solution and left to incubate for 24 h. Subsequently, the cells underwent irradiation using an 808-nm laser for different durations: 0, 4, 6, 8, and 10 min. After the treatment, the cells were further incubated in a CO_2_ incubator for an additional 24 h and then examined with Hoechst 33342/PI counterstaining. The control group and those irradiated with a laser for 4 min exhibited a substantial number of healthy cells emitting a blue signal (Fig. 5L and M), whereas the samples treated with the laser for 6 and 8 min displayed moderately healthy cells with some dead cells. Samples irradiated for 10 min exhibited over 95% of cells in a dead state due to the ROS generated by the nanocomposite (Fig. 5P).

The generation of ^1^O_2_ species by the MPB nanocomposite in vitro was validated using dichlorodihydrofluorescein diacetate (DCFH-DA). This process involves the conversion of nonfluorescent DCFH into fluorescent dichlorodihydrofluorescein (DCF) through oxidation by ^1^O_2_. Freshly prepared MDA-MB-231 cells were divided into 4 groups and cultured on a plate for 24 h. The 4 groups were treated as follows: Group I remained untreated, group II was treated with H_2_O_2_, group III was treated with MPB nanocomposite, and group IV was treated with a combination of MPB nanocomposite and H_2_O_2_. The treated cells were then further incubated for an additional 4 h, and 1 ml of DCFH-DA was added to each group. After a 10-min staining period, group II to IV samples were subjected to an 808-nm laser (1.0 W/cm^2^) for 10 min, and the cellular images were examined using CLSM, as depicted in Fig. 5G to K. No fluorescence emission was observed initially without laser irradiation (time 0 s, Fig. 5G). Throughout the treatment time, the cells were continuously monitored at 10-s intervals to identify which cell emits the strongest green fluorescence (Fig. 5H to K and Movie S1).

Most experiments presented in Figs. 4 and 5 involved 11 min of laser irradiation. However, Fig. 5G to P features shorter irradiation times, ranging from 0 to 40 s. The shorter irradiation durations were used to capture early-stage data on the generation of singlet oxygen species to provide insights into the initial ROS activity. Figure 5L to P presents Hoechst 33342/PI staining results at various irradiation times (0, 4, 6, 8, and 10 min), which were used to evaluate how different irradiation durations affect cell viability. Figure 4O illustrates the effects of concentration and laser power on the PTT performance of MPB nanocomposites, whereas Fig. 5F represents in vitro post-photodynamic study MTT assay of anti-EGFR-MPB nanocomposite (250 μg/ml)-incubated MDA-MB-231 cells on 808-nm laser exposure at 1.0 W/cm^2^.

### Photoacoustic imaging

**Fig. 6.**
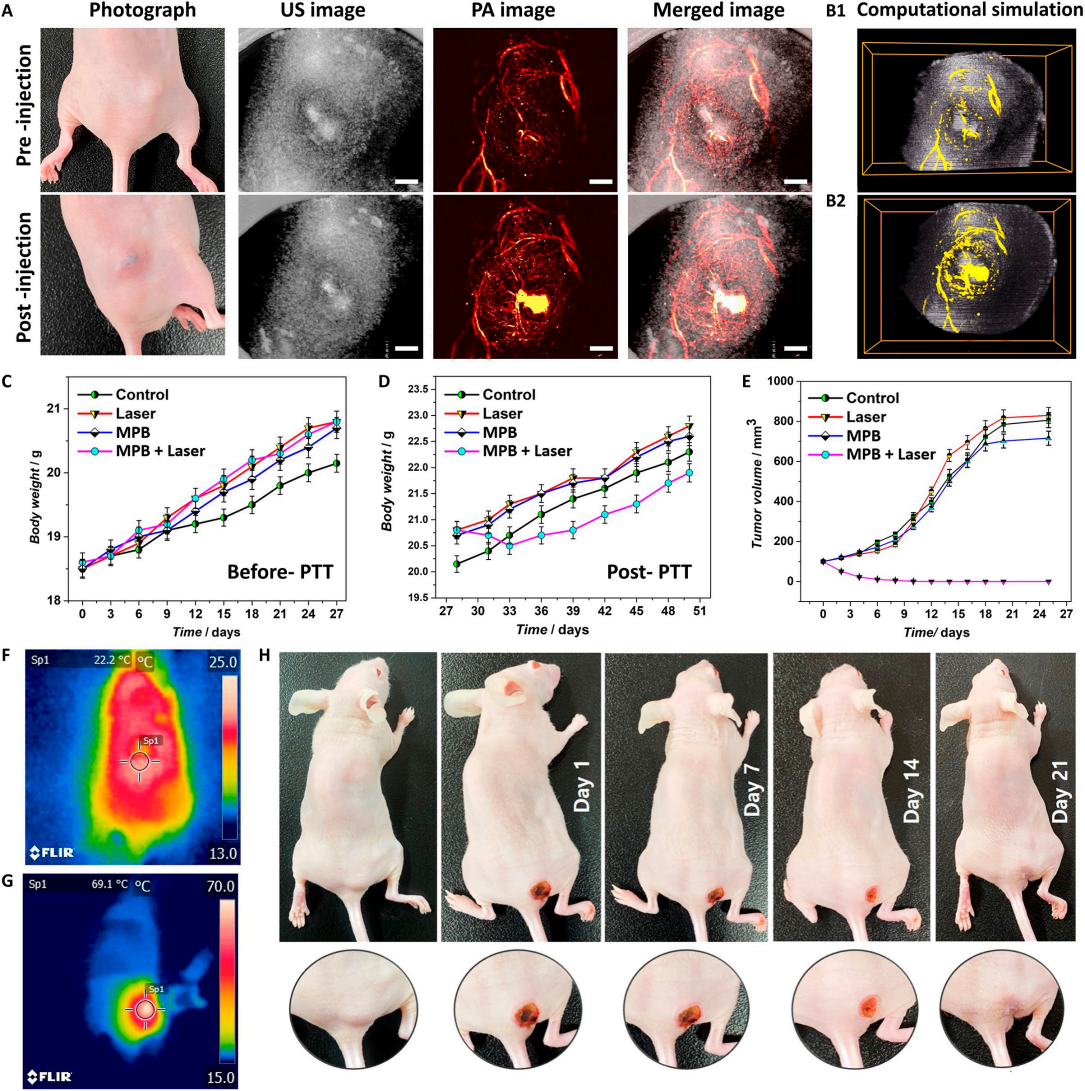
(A) In vivo 3D PAI of tumor tissues of nude mice before and after the injection of anti-EGFR-MPB nanocomposite. The photoacoustic (PA) imaging was carried out utilizing a PAUS system spanning 532 to 1,000 nm, applying filters in the range of 625 to 1,000 nm. The 3D images constructed using experimental photoacoustic microscopy (PAM) data are presented in Movie S2. (B1 and B2) In vivo 2D PAI images of the tumor tissues in MDA-MB-231 tumor-bearing nude mice. (C) Initial mouse body weights prior to PTT treatments. (D) Mouse body weights after PTT treatments. (E) Evolution of tumor volume in mouse groups following PTT treatments. Data are presented as mean ± SD (*n* = 3, *P < 0.05). (F and G) IR thermal images of a selected mouse before and during PTT treatment (7 min of laser exposure). (H) Illustrative images of nude mice carrying MDA-MB-231 tumors on day 0, on the commencement of photothermal treatment (day 1), and progression until complete healing (day 21).

For PAI, a solution of anti-EGFR-MPB composite at a concentration of 250 μg/ml was intravenously administered to mouse models targeting MDA-MB-231 tumors at a dose of 100 μl (Fig. 6C). An experimental study was conducted before and after injection of anti-EGFR -MPB using 2 filter modes to image blood vessels (532 to 1,000 nm) and the nanocomposite to evaluate the impacts of anti-EGFR-MPB (625 to 1,000 nm) on tumor-specific binding affinity. For this purpose, 2 distinct filters were used to scan the entire tumor region in the pre-injection stage, and US imaging was performed in the tumor region for better image analysis. Owing to the absorption of hemoglobin (from blood) at 532 nm, only blood vessels in the tumor location were observed (Fig. 6A). On the contrary, the 625-nm filter entirely removed the blood hemoglobin absorbance signal. Injection of the anti-EGFR-MPB solution illuminated a distinct region within the tumor and blood vessels. Subsequent scanning with the 625-nm filter precisely depicted the tumor’s position, form, and dimensions. Finally, PAI and US images were analyzed and combined (PAUS) to produce 3-dimensional (3D) images with precise tumors and surrounding blood vessel locations.

### In vivo PTT

The Pukyong National University, Busan, South Korea, authorized the institutional policies and procedures for animal care services, which were followed in all animal experimentation performed in this work (permit number: PKNUIACUC-2022-16) (see the Supplementary Materials for animal model study). The mice under investigation were divided into 5 groups. Among then, the mice of group I were used as healthy controls. The mice of group II served as negative controls (with tumors but no treatment). The mice of groups III, IV, and V are tumor induced. However, the mice of group III were treated with laser without nanocomposite administration. On the other hand, the mice of group IV were treated with MPB nanocomposite (administered by injection) without laser exposure. Finally, the mice of group V underwent laser therapy after MPB injection. Group III animals did not experience any remarkable heat generation when exposed to the laser alone (Table S2). The average body temperature of these mice was 23.4 °C, and the room temperature was 18 ± 2 °C. Following exposure to a 1.0 W/cm^2^ laser (808 nm) for 7 min, the maximum temperature increase observed was 1.7 °C, resulting in a temperature of 25.1 °C (final body temperature), as the laser alone was not able to heat the target tumor location in the absence of the nanocomposite. The group IV mice were injected with nanocomposite, but not treated with laser. However, when 250 μg/ml of MPB solution was intratumorally injected (group V animals) and then exposed to a laser with an intensity of 1.0 W/cm^2^ at a wavelength of 808 nm (for 7 min), their body temperature rose from 22.2 to 69.1 °C as can be observed from the IR thermal images presented in Fig. 6F and G. Following PTT, the temperature in the tumor area was reduced to 27.4 °C, nearing the normal body temperature of the mouse. The elevated temperature in the tumor region effectively led to the successful ablation of tumors in vivo. The safety of the therapy was demonstrated by the fact that none of the animals died even after 21 d of photothermal treatment. In addition, neither the behavior nor the body weight of the mice significantly changed due to photothermal shock. ICP-MS (discussed later) did not reveal detectable toxicity or deadly effects of accumulated MPB nanocomposite in their vital organs [37]. After the treatment, continuous monitoring of all mice was conducted for a complete 4-week period to verify the total disappearance of the photothermal impacts (Fig. 6D, E, and H).

The MDA-MB-231 cancer cells required 4 weeks to develop a functional tumor within the mouse's body following injection, as depicted in Fig. 6C. On the 28th day after the injection of tumor cell, we initiated the PTT treatment. Throughout the treatment period, the mice received no additional medications beyond their regular food and drink. We continuously monitored the treatment’s progress, observing the healing of the tumor-affected area and the absence of further tumor growth in the mice. We also regularly measured the tumor’s weight and volume, as illustrated in Fig. 6C to E.

At regular intervals, we measured the body weight of each mouse in the experiment. All groups exhibited consistent weight increases during the initial 27 d following tumor induction, as depicted in Fig. 6C. The treatment results revealed a significant reduction in tumor growth and no recurrence in the group of mice treated with both laser and nanocomposite, in contrast to the control group and other treatment groups, where tumors continued to grow (Fig. 6D). This observation was substantiated by tracking changes in tumor volume and the weight of the animals, with the control group experiencing a notable increase in weight due to tumor growth (Fig. 6E). Group V mice undergoing photothermal treatment initially showed a rapid drop in weight, but 1 week later, the group resumed its normal weight gain (Fig. 6D).

### Biodistribution of composite MPB nanocomposite

**Fig. 7.**
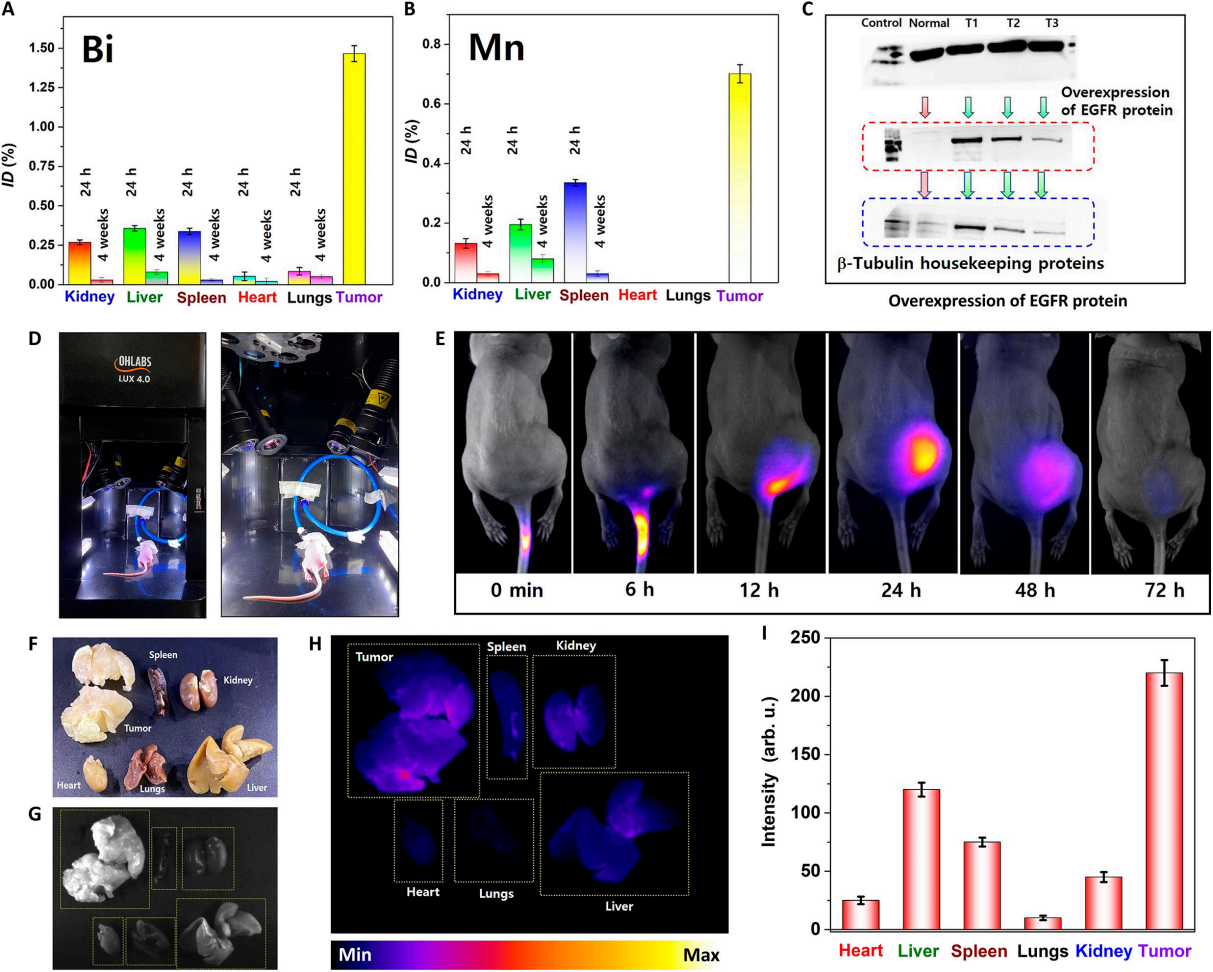
Biodistribution of (A) Bi and (B) Mn in various organs (spleen, heart, kidney, liver, and lungs) and tumor tissues of mice subjected to treatment with anti-EGFR-MPB nanocomposite. (C) Western blot protein assay results indicating the overexpression of anti-EGFR in comparison to normal cells. (D and E) Fluorescence imaging of anti-EGFR-MPB, monitored after tail vein injection of anti-EGFR-MPB nanocomposite. (F to H) Fluorescence images of organs harvested 72 h after intertumor tail vein injection. (I) Comparative fluorescent intensities in different organs.

In vitro cellular uptake was evaluated by an ICP-MS study (Fig. S6C). Mice in the treatment groups were euthanized at 24-h and 4-week intervals to examine the presence of MPB accumulation in their organs. The harvested organs were divided into 2 groups and subjected to an 8-h treatment with HNO_3_ to facilitate lysis. Bi and Mn concentrations in each group were quantified using ICP-MS. The results revealed substantial levels of Bi and Mn at the 24-h mark following tumor treatment, as shown in Fig. 7A and B. The estimated cumulative quantities of Bi and Mn per gram of tumor tissue after 24 h were approximately 1.46% and ~0.7%, respectively.

The nanocomposite was administered intravenously rather than directly into the tumor, leading to their dispersion within the bloodstream and distribution throughout various organs in the body. Consequently, the liver, spleen, and kidneys exhibited the highest Bi accumulation (0.35%, 0.33%, and 0.26% ID/g after 24 h and 0.08%, 0.03%, and 0.03% ID/g after 3 weeks), whereas the lungs and heart showed the following Bi accumulation: 0.08% and 0.05% ID/g at 24 h after treatment. After 3 weeks, the examination of nanocomposite distribution throughout the body indicated that they had largely been cleared from the body’s systems, with only minimal residual amounts in the lungs and heart: 0.05%, 0.02%, and 0.01% ID/g, respectively.

Analysis of the ICP results for Mn revealed that the spleen, liver, and kidneys exhibited the highest accumulation of the nanocomposite, with values of 0.33%, 0.19%, and 0.13% ID/g after 24 h, and 0.03%, 0.08%, and 0.03% ID/g after 3 weeks, respectively. In contrast, the lungs and heart showed no detectable nanocomposite accumulation at either the 24-h or 3-week time points. Importantly, the minimal presence of nanocomposite in the organs resulted in no observed adverse or fatal effects.

### Fluorescence imaging study of anti-EGFR-MPB nanocomposite

The in vitro study of the anti-EGFR-MPB nanocomposite confirmed its high-intensity fluorescence properties at a concentration of 250 μg/ml (Fig. S7). This study was extended to an in vivo BALB/c nude mouse model, where the nanocomposite was administered via tail vein injection for targeted accumulation in the tumor region. The composite MPB NPs conjugated to the antibody, with a conjugation efficiency of approximately 87.02%, as estimated using fluorescence spectroscopy (Figs. S8 to S10).

The nanocomposite solutions were injected 3 times in sequential volumes of 100, 75, and 50 μl at 60-min intervals. Fluorescence images of tumor-bearing mice were captured at various time points (6, 12, 24, 48, and 72 h) until no further fluorescence signals were detected from the mouse's body (Fig. 7C to E). These fluorescence properties confirmed the successful conjugation of the nanocomposite to the tumor region, with fluorescence intensity remaining consistent until the 48-h post-injection mark. Beyond 48 h, the fluorescence intensity decreased due to the metabolic activity of the cellular system, and by the 72-h interval, no signal was detectable in the mouse’s body.

Following 72 h of imaging, the mice were euthanized, and their organs were harvested to investigate the organ-accumulated fluorescence signals (Fig. 7F to H). The highest intensity of the nanocomposite system was observed during the first 24 h of imaging, considered the optimal window for in vivo photothermal treatment. Notably, the tumor exhibited the highest fluorescence intensity, demonstrating successful targeting by the nanocomposite through the anti-EGFR affinity moieties. Among the harvested organs, the liver, spleen, and kidneys exhibited moderate fluorescence signals, while other organs showed no significant or very weak signals (Fig. 7I).

### Hemolysis study

**Fig. 8.**
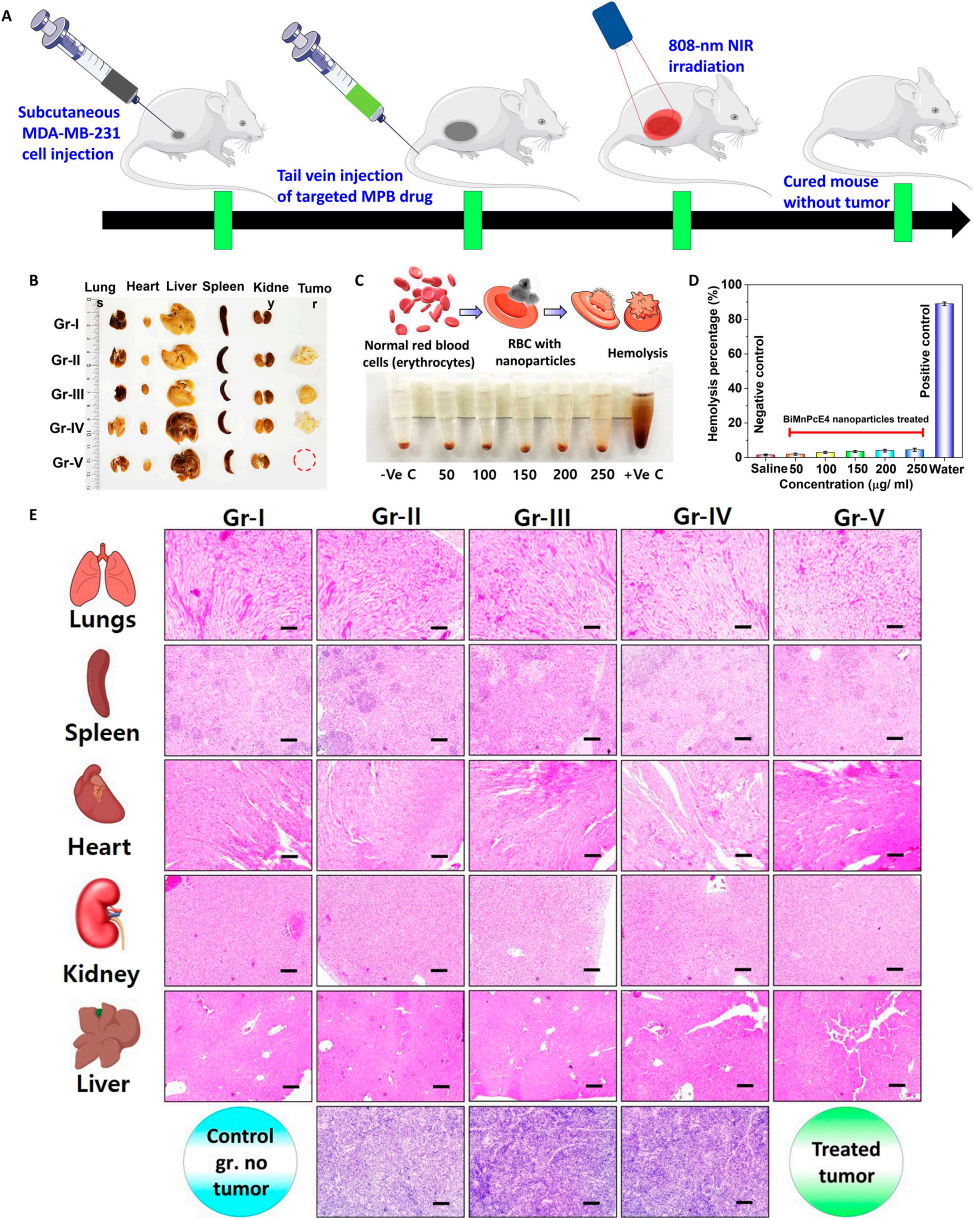
(A) Digital images of organs (heart, liver, lungs, kidney, and spleen) and tumor recovered from posttreatment mouse model. (B to D) Schematic and results of hemolysis study of anti-EGFR-MPB nanocomposite. (E) H&E-stained images of organs (lungs, spleen, heart, kidney, liver, and tumor) after PTT treatments (20× magnification; scale bars, 50 μm).

Blood samples stabilized with heparin were collected from nude mice and then centrifuged to prepare them for the hemocompatibility assay (Fig. 8B and C). A sample of red blood cells (RBCs) was collected, diluted with PBS, and then exposed to MPB nanocomposite at concentrations of 50, 100, 150, 200, and 250 μg/ml in a 1.1-ml PBS solution. Distilled water was employed as a positive control, while a medical saline solution served as a negative control. Each solution was mixed using a vortex, kept for 4 h at 37 °C, and then centrifuged for 6 min at 5,000 rpm. The absorbance of individual samples was assessed at a wavelength of 541 nm using a Tecan Infinite F50 microplate reader. Hemolysis of RBCs in each sample was determined using the following equation:

$$\text{Hemolysis}\;\left(\%\right)=\frac{{\text{Abs}}_{\text{Sample}}-{\text{Abs}}_{\text{Negative control}}}{{\text{Abs}}_{\text{Positive control}}-{\text{Abs}}_{\text{Negative control}}}$$ (4)

As part of in vivo cancer therapy, the anti-EGFR-MPB nanocomposite was injected to the BALB/c nude mouse through the tail vein area. It is essential to investigate the compatibility of the injected nanocomposite with blood. The results presented in Fig. 8B and C reveal that anti-EGFR-MPB exhibited a hemolytic activity of approximately 4.17% at a high concentration of 250 μg/ml, indicating the exceptional blood compatibility of the synthesized nanomaterials (Fig. 8C)

### Western blotting tests

The MDA-MB-231 cell line is a widely employed human breast cancer cell line known for its overexpression of EGFR, a protein pivotal in cell growth and proliferation. In our Western blotting study (Fig. 7C), we assessed the expression levels of EGFR in MDA-MB-231 cells treated with the anti-EGFR-MPB nanocomposite compared to untreated normal cells. The anti-EGFR-MPB nanocomposite, designed to specifically target EGFRs on cancer cells, was synthesized and coupled with an anti-EGFR antibody. By binding to the EGFR, these nanocomposites can deliver therapeutic agents directly to the cancer cells, facilitating the selective elimination of cancer cells while safeguarding normal cells.

### Histological analysis

In vivo experimental studies were conducted over 7 weeks, spanning from the induction of the tumor to the photothermal treatment (Fig. 8A). Histological analysis conducted after the completion of the PTT study provided valuable insights into the effects of the treatment on both primary organs and tumor tissues. Each stage of the in vivo investigation, from tumor induction to photothermal treatment, spanned 7 weeks. Upon examination of tissues from primary organs (lungs, spleen, heart, kidney, and liver), cross-sections stained with H&E revealed no abnormal morphology or signs of damage (Fig. 8A). The organs appeared normal in color and structure across all experimental groups (I to V) (Fig. 8D), indicating that treatment with the anti-EGFR-MPB nanocomposite did not cause harm to the body. The findings were further supported by the absence of tissue damage, abnormal behavior, or alterations in body weight observed during the study period.

In contrast, the histological sections of tumor tissues revealed significant changes associated with PTT (Fig. 8D). Animals treated with laser irradiation and anti-EGFR-MPB (group IV) showed notable inhibition of tumor growth, with no malignant tissues detected after treatment. Healthy cells with distinct nuclei were observed in the surrounding tissues, indicating successful tumor inhibition and the absence of recurrence. In comparison, untreated tumors in groups II and III exhibited cells lacking differentiation and deeply stained granular nuclei, highlighting the effectiveness of the anti-EGFR-MPB nanocomposite in inhibiting tumor growth.

## Discussion

The anti-EGFR-MPB nanocomposite represents a significant advancement in precision cancer therapy, particularly for breast cancer treatment. XRD analysis confirmed the successful integration of Bi and Mn components into the nanocomposite, maintaining its crystalline structure, which is essential for biomedical applications. UV-vis and FTIR spectroscopy demonstrated the nanocomposite’s broad light absorption and structural stability, crucial for imaging and therapeutic functions. DLS and zeta potential studies validated its stability and dispersion in biological medium, confirming effective bioconjugation and enhancing its suitability for targeted therapeutic applications. Further analysis using XPS and TEM confirmed the surface composition and structural integrity, indicating the nanocomposite’s potential for precise cancer cell targeting and treatment. The strong optical absorption and efficient photoacoustic signal generated by anti-EGFR-MPB particles highlight their potential in PAI. MPB particles exhibit a high molar extinction coefficient in the NIR range, making them excellent laser light absorbers. Utilizing an 808-nm NIR laser enabled deep tissue penetration of excitation signals, while MPB particles as biomarkers enhanced the contrast and spatial resolution of tumor images. High-resolution tumor images obtained by combining US and PAI allowed precise targeting of laser signals for tumor ablation, as demonstrated in Movie S2.

The photothermal performance of the anti-EGFR-MPB nanocomposite, with its efficient light-to-heat conversion capabilities, was demonstrated under 808-nm laser irradiation, reaching temperatures sufficient for hyperthermia treatment. Ideal temperatures for in vivo hyperthermia treatment typically remain between 42 and 47 °C. The PTT study results indicate that the fabricated nanocomposite is highly effective for hyperthermia treatment, even at a concentration of 0.5 μg/ml. In contrast, the control sample of PBS solution only reached a temperature of 28.2 °C, indicating its unsuitability for hyperthermia application. The photothermal performance and IR thermal imaging of MPB solutions showed a strong correlation, establishing that the optimal therapeutic dose of the MPB nanocomposite is 250 μg/ml at 1.0 W/cm^2^ laser power for cancer treatment.

This performance, combined with minimal cytotoxicity in the absence of laser irradiation, underscores the nanocomposite's efficacy for selective cancer cell ablation. The in vivo studies reinforced these findings, showing significant tumor ablation in mice treated with the anti-EGFR-MPB nanocomposite and laser, with no adverse effects or recurrence. Biodistribution and clearance studies indicated minimal long-term toxicity, while fluorescence imaging confirmed effective tumor targeting. The study concluded that photothermal cancer therapy using the nanocomposite and administered doses was safe, nonlethal, and highly effective. Hemocompatibility assays and molecular analyses, including Western blotting and histological examinations, further validated the nanocomposite's safety and targeted therapeutic action. The Western blotting results revealed a significant decrease in EGFR expression in MDA-MB-231 cells treated with the anti-EGFR-MPB nanocomposite compared to untreated normal cells. This reduction in EGFR expression suggests effective targeting and binding of the nanocomposite to the EGFR cancer cell surfaces, leading to potential therapeutic effects. In this study, the anti-EGFR antibody played a pivotal role by targeting and inhibiting the Ras–Raf–MEK [mitogen-activated protein kinase (MAPK) kinase]–MAPK signaling pathway. This pathway is crucial in regulating cell growth, proliferation, and cell survival. Anti-FGFR (fibroblast growth factor receptor) is a prime targeting agent for therapeutic intervention in cancers where it is dysregulated, often due to overactive EGFR signaling. EGFR is a cell surface receptor that, upon activation by ligands such as EGF, initiates a cascade of intracellular signaling events through Ras, Raf, MEK, and MAPK. This cascade ultimately leads to the activation of genes involved in cell proliferation, differentiation, and survival. The anti-EGFR antibody binds specifically to EGFR, thereby preventing its activation by ligands. By doing so, it effectively blocks the initiation of the Ras–Raf–MEK–MAPK pathway downstream of EGFR. Consequently, this inhibition disrupts the signaling required for cancer cells to grow and proliferate uncontrollably. This interruption is crucial for reducing cancer cell proliferation and promoting cell death (apoptosis). Additionally, targeted NPs will further generate hyperthermia and ROS by the combined effect of PTT and PDT. This could include tumor shrinkage, prolonged progression-free survival, or improved patient outcomes, all indicative of effective pathway inhibition. The selective targeting of cancer cells by the nanocomposite indicates minimal impact on normal cells, highlighting the specificity and potential clinical relevance of this approach.

## Conclusion

The development of targeted dual image-guided phototherapy nanocomposite marks a significant advancement in cancer treatment. We demonstrated the exceptional contrast efficiency and high photothermal activity of the anti-EGFR-MPB nanocomposite in mouse models with triple-negative breast cancer. Through its biomimetic design, the nanocomposite achieves precise cancer cell targeting via anti-EGFR conjugation, while dual-modality imaging enables real-time treatment visualization. Under 808-nm laser irradiation, the nanocomposite demonstrates synergistic PDT and PTT effects, further enhanced by the presence of Mn^2+^ ions, which promote O_2_ generation from H_2_O_2_, thus alleviating tumor hypoxia and enhancing PDT performance. Notably, the nanocomposite offers a cost-effective alternative to gold-based materials, exhibiting similar efficiency (~33.8%) in converting light into heat for photothermal treatment. In vivo fluorescence imaging confirms tumor site accumulation of the nanocomposite, facilitated by the enhanced permeability and retention (EPR) effect, and metabolic pathways in major organs, thus enabling image-guided antitumor treatment. Our findings underscore the strong anticancer efficacy of PTT/PDT guided by PAI, both in vitro and in vivo, showcasing selective cancer cell destruction while minimizing damage to healthy cells. The targeted approach demonstrated in this study holds promise for enhancing cancer therapy precision and efficacy. However, further investigation is needed to evaluate the safety, efficacy, and long-term effects of this nanocomposite. Our study suggests an effective treatment regimen of 250 μg/ml of anti-EGFR-MPB nanocomposite combined with 1.0 W/cm^2^ laser irradiation for 7 min for treating triple-negative breast cancer.

## Data Availability

Data will be made available on request.

## Supplementary Materials

Supplementary 1 Supplementary Text Figs. S1 to S10 Tables S1 and S2 Movies S1 and S2 References
