# Supplementary material for: Precision Cancer Therapy Enabled Anti-Epidermal Growth Factor Receptor-Conjugated Manganese Core Phthalocyanine Bismuth Nanocomposite for Dual Imaging-Guided Breast Cancer Treatment
Source: Biomater Res. 2024 Nov 7;28:0092. doi: 10.34133/bmr.0092 (PMC11542904; doi:10.34133/bmr.0092)
Supplement: Supplementary 1 — Supplementary Text Figs. S1 to S10 Tables S1 and S2 Movies S1 and S2 References [file bmr.0092.f1.zip › Supplemental Material Clean Version.docx]

**Supporting Material**

Precision Cancer Therapy Enabled Anti-EGFR-MPB Nanocomposite for Dual Imaging-Guided Breast Cancer Treatment

Sudip Mondal,^1#^ Sumin Park,^2#^ Van Tu Nguyen,^2 @^ Vu Hoang Minh Doan,^2^ Jaeyeop Choi,^3^

Cao Duong Ly,^2^ Duc Tri Phan,^2 ¥^ Thi Thuy Truong,^2^ Tan Hung Vo,^2^ Dinh Tuan Nguyen,^2^ Umapada Pal,^4^ Byeongil Lee,^1,2,3,5 †^ Junghwan Oh^1,2,3,5,6^*

^1^ Digital Healthcare Research Center, Pukyong National University,

Busan, 48513, Republic of Korea

^2^ Industry 4.0 Convergence Bionics Engineering, Department of Biomedical Engineering, Pukyong National University, Busan 48513, Republic of Korea

^3^ Smart Gym-Based Translational Research Center for Active Senior’s Healthcare, Pukyong National University, Busan 48513, Republic of Korea

^4^ Institute of Physics, Autonomous University of Puebla, Puebla, Pue. 72570, Mexico

^5^ Department of Smart Healthcare, Pukyong National University, Busan, 48513, Republic of Korea

^6^ Ohlabs Corp., Busan 48513, Republic of Korea

* Corresponding Author: [jungoh@pknu.ac.kr](mailto:jungoh@pknu.ac.kr), Tel: +82-51-629-5771, Fax: +82-51-629-5779

^†^ Co-corresponding Author: [bilee@pknu.ac.kr](mailto:bilee@pknu.ac.kr)

^#^ Contributed equally to this work

^@^ Current Affiliation: Biomedical Engineering, Duke University, Durham, NC, 27708, USA

^¥^ Current Affiliation: The School of Electrical and Electronic Engineering, Nanyang Technological University, 639798, Singapore

^2#^ Current Affiliation: Department of Biomedical Engineering, University of Michigan,

Ann Arbor, MI, USA

**Chemicals and reagents**

All of the chemicals used in this research were procured from Sigma Aldrich, St. Louis, Missouri, USA. The Dulbecco's modified Eagle's medium (DMEM), fetal bovine serum (FBS), antibiotic, trypsin, and phosphate-buffered saline (PBS) were obtained from HyClone in South Logan, Utah, USA. Deionized water with a resistivity exceeding ρ > 18.2 MΩ∙cm (at 25 °C) was obtained from a Millipore deionizer and employed throughout the study.

**Synthesis of 1,8,15,22-tera-(3-(4-ethylcarboxyl-phenoxy)) phthalocyanine manganese (II) (MP)**

Initially, 0.50 g (3.23 mmol) of 3-(4-ethylcarboxyl-phenoxy) phthalonitrile, 0.20 g (1.62 mmol) of manganese (II) chloride, and 1 mL of 1,8-Diazabicyclo 5.4.0 undec-7-ene (DBU) were mixed with 35 mL of ethylene glycol. The resulting mixture was refluxed at 195 ºC for 10 hours under a nitrogen atmosphere. After the 10-hour reaction period, the solution was mixed with 30 mL of 0.1 M NaOH solution and filtered using a syringe filter (Minisart 0.2 μm). Subsequently, the dissolved product was precipitated by adding 2.5 mL of 1 M HCl solution. The product was crystallized from acetonitrile followed by separated and washed multiple times with deionized water. Finally, the product MP was dried at 40°C for 8 hours and stored at room temperature for further synthesis process.

**Synthesis of MPB nanocomposite and their PEG functionalization**

To begin with, 53.1 mg of Bi(NO_3_)_3_∙5H_2_O and 104.2 mg of tetraoctylammonium bromide (TOAB) were dissolved in 10 mL of ethylene glycol under magnetic stirring for 1 hour. After that, about 207.2 mg of phthalocyanine manganese mixed with 5 mL of tetrahydrofuran (THF) was added to the mixture rapidly, and stirring was continued for 1 hour. Then the reaction mixture was placed on an ice-cold water bath and 72.1 mg of NaBH_4_ was added rapidly. The red solution turned black after 1 hour. The reaction mixture was stirred for another 30 minutes, and the product was separated by centrifugation. After centrifugation, the product was collected, washed with anhydrous ethanol for 4 times, and then dried under vacuum. The synthesized MPB nanocomposite (10 mg) was dispersed in 1 mL of THF to increase its water stability. The colloidal solution was then added dropwise to the DSPE-PEG3000 solution, which was prepared by dispersing DSPE-PEG3000 (20 mg) in deionized water/THF (v/v, 5:1, 15 mL) mixture. Finally, the DSPE-PEG3000 modified MPB nanocomposite was purified through successive dialysis (molecular weight cut-off: 3000 Da).

**Characterization**

Physicochemical characteristics of the nanomaterials were analyzed using energy-dispersive X-ray spectroscopy (EDS) and field emission transmission electron microscopy (FETEM, JEM-2100F; JEOL, Tokyo, Japan). Absorbance spectra of the synthesized MPB were recorded using an ultraviolet-visible (UV-vis) spectrophotometer (Genesys 30S) from Thermo Fisher Scientific, Waltham, Massachusetts, USA. X-ray photoelectron spectroscopy (XPS) was performed using an AXIS Supra electron spectrometer from Kratos Analytical Ltd. in Manchester, UK. The binding energies (BEs) of the elements were calculated to a precision of 0.1 eV, and peaks were analyzed with the help of ESCApeTM software from Kratos Analytical Ltd. Fourier-transform infrared (FTIR) spectroscopy (JASCO FRIT 4100) was used to determine the functional groups in MPB. Surface zeta potential (ZP) and dynamic light scattering (DLS) studies were conducted using an electrophoretic light scattering spectrophotometer (ELS-8000) from Otsuka Electronics Co. Ltd in Osaka, Japan. An inductively coupled plasma mass spectrometer (ICP-MS; PerkinElmer-NexION 300D, PerkinElmer, Inc., USA) was used to estimate the nanostructures' Bi/Mn ion concentration.

The photothermal effects were studied using a near-infrared laser of 808 nm emission from Changchun New Industries Optoelectronics Technology in Changchun, China. Thermal images were captured using a digital infrared thermal imaging camera from FLIR Systems Inc. in Portland, Oregon, USA. Both *in vitro* and *in vivo* photoacoustic imaging were performed using a photoacoustic microscopy (PAM) system (FPAM-v1) from Ohlabs Inc. in Busan, South Korea. Raw data were processed in MATLAB to generate photoacoustic images.

**Biological activity and in vitro cytotoxicity**

The MDA-MB-231 human breast cancer cell line was procured from the Korean Cell Line Bank (KCLB) for cell culture research. These cells were maintained in Dulbecco's modified Eagle's medium (DMEM; Hyclone), supplemented with 1% penicillin/streptomycin antibiotics and 10% fetal bovine serum (FBS). The cultured cell plates were incubated at 37°C with 5% CO_2_. To assess the toxicity of the synthesized MPB nanocomposites, the MTT (3-(4,5-dimethylthiazol-2-yl)-2,5-diphenyltetrazolium bromide) test was conducted. Initially, 10^4^ MDA-MB-231 cells were seeded in a 96-well plate and cultured for 24 hours. Subsequently, the cells were exposed to various concentrations of MPB nanocomposites for 24 hours, specifically 50, 100, 150, 200, 250, and 300 µg mL^-1^. After a 6-hour incubation, the spent medium was removed from each well, and a fresh medium containing the MTT solution was added. The formazan crystals generated during the MTT reaction were then dissolved using dimethyl sulfoxide (DMSO), and the color intensity was measured at 570 nm using a Tecan Infinite F50 microplate reader.

**Western Blotting tests**

Protein Extraction:

Proteins were extracted from MDA-MB-231 breast cancer tumor cells, both untreated and treated with anti-EGFR-MPB nanocomposite, using a lysis buffer containing protease inhibitors. The cells were washed and lysed in a buffer solution composed of 50 mM Tris-Cl (pH 7.5), 150 mM NaCl, 1 mM DTT, 0.5% NP-40, 0.1% SDS, 1% Triton X-100, and 1% deoxycholate. The lysate was incubated on ice for 30 minutes and then centrifuged at 4°C for 25 minutes.

Protein Quantification: The supernatant containing protein was transferred to a new tube, discarding the sediment. Total protein concentration was then determined using albumin standard reagent and Bradford reagent on an ELISA reader, with samples protected from light. The obtained data were then analyzed in Excel to determine the exact amount of protein required for western blotting.

SDS-PAGE: The protein samples were loaded onto a polyacrylamide gel for electrophoresis, to separate the proteins by their size.

Transfer: The extracted protein lysate was subjected to Western blot analysis using a 12% running gel and a 5% stacking gel. After separating the protein mixture in the electrophoresis tank, the gel membrane was transferred to the positive electrode and allowed to run between filter paper and a fiber pad.

Blocking: Following the transfer, the membrane was blocked using 1X PBST and 5% skim milk to prevent non-specific binding.

Antibody incubation: The membrane was incubated overnight at 4°C with a primary antibody against EGFR conjugated with manganese core phthalocyanine bismuth composite.

Secondary Antibody Incubation: After washing, the membrane was incubated with a secondary antibody conjugated to an enzyme (e.g., horseradish peroxidase) for 1-2 hours at room temperature.

Visualization: The protein of interest was visualized by adding a chemiluminescent substrate that reacts with the HRP-conjugated secondary antibody.

Detection: An image of the membrane was captured using a chemiluminescence imaging system, and the band intensity was quantified using densitometry software.

**Animal model**

Pukyong National University in Busan, South Korea, approved and adhered to institutional policies and procedures for animal care services, as documented under the reference number PKNUIACUC-2022-16. A group of female BALB/c nude mice, weighing approximately 18.9 ± 0.8 grams and aged between six and seven weeks, was procured from Orient Bio Inc. in Seongnam, Republic of Korea. To establish tumor xenografts, 10^6^ MDA-MB-231 cells suspended in 100 µL of PBS were subcutaneously injected into the flank of each mouse. Tumor growth was closely monitored and maintained until the tumors reached a volume of approximately 100 mm^3^, which was measured using digital calipers. Subsequently, the animals were divided into five experimental groups, with a total of 20 animals participating, as outlined in Supporting Information Table S1. A tail vein-mediated injection of a 100 mL solution containing MPB nanocomposite (250 µg mL^-1^) was administered to the tumor-bearing mice. These mice were then housed in an appropriate environment and constantly observed. Tumors in each mouse were subjected to a 7-minute radiation treatment using an optical fiber connected to an 808 nm laser diode. The study involved four groups of mice:

1. A control group of mice without tumors that did not receive any treatment.
2. Mice with tumors that did not receive any treatment.
3. Mice with tumors exposed only to the laser.
4. Mice with tumors injected with the nanocomposites referred to as MPB but not exposed to the laser.
5. Mice with tumors injected with MPB and exposed to the laser.

A probe thermometer was inserted into the center of each tumor before radiation treatment. The tumor's temperature was monitored at one-second intervals throughout the course of the radiation treatment.

**Table S1.** *In vivo* study model with five divided groups

| Groups | Treatment | Total  animals | Experimental conditions |
| --- | --- | --- | --- |
| I | Control (without tumor) | 4 | Suspension volume: 100 µL  Duration of irradiation: 7 min. Power density: 1.0 W∙cm^-2^  Wavelength: 808 nm  Cancer cells: MDA-MB-231 |
| II | Only Tumor (no treatment) | 4 |  |
| III | Only laser treated | 4 |  |
| IV | Only Anti-EGFR-MPB treated (no laser) | 4 |  |
| V | Treated with Anti-EGFR-MPB + Laser | 4 |  |
| Total of animals studied | | 20 |  |

**Photoacoustic imaging (PAI) & in vivo photothermal therapy**

This PAM setup utilized a Q-switched diode-pumped solid-state laser (model SPOT-10-100-532; Elforlight, Daventry, UK) along with a commercially available focused transducer (model V324-SM; Olympus, USA). A focused illumination beam was precisely aligned with the transducer using a custom-made optical acoustic beam combiner. Subsequently, the photoacoustic (PA) signals were amplified and captured by a digitizer (model PXI-5124; National Instruments, USA) with a sampling rate of 200 MS/s samples (Million Sample/s). The gathered PA signals were then processed using MATLAB to convert them into PA images. The PAI on tumor-bearing mice was performed using the PAM system at wavelengths of 532, 532–1000 nm, and 625–1000 nm.

**Fluorescence imaging**

The used fluorescence imaging system (LUX 4.0; OHLABS, Busan, Republic of Korea) is an upgraded version of LUX 3.0, as introduced in our earlier study. The system comprises four channels, each associated with a distinct set of excitation and emission modules. The synthesized MPB nanocomposite were excited with a 635 nm excitation wavelength, and the emission spectra were recorded at 703 nm. To obtain images, the emitted signals from the sample were filtered and being captured by the imaging module. Subsequently, the raw data were processed using the system's proprietary software to generate fluorescence images.

**Hydrodynamic size and dispersibility study of MPB nanocomposites**


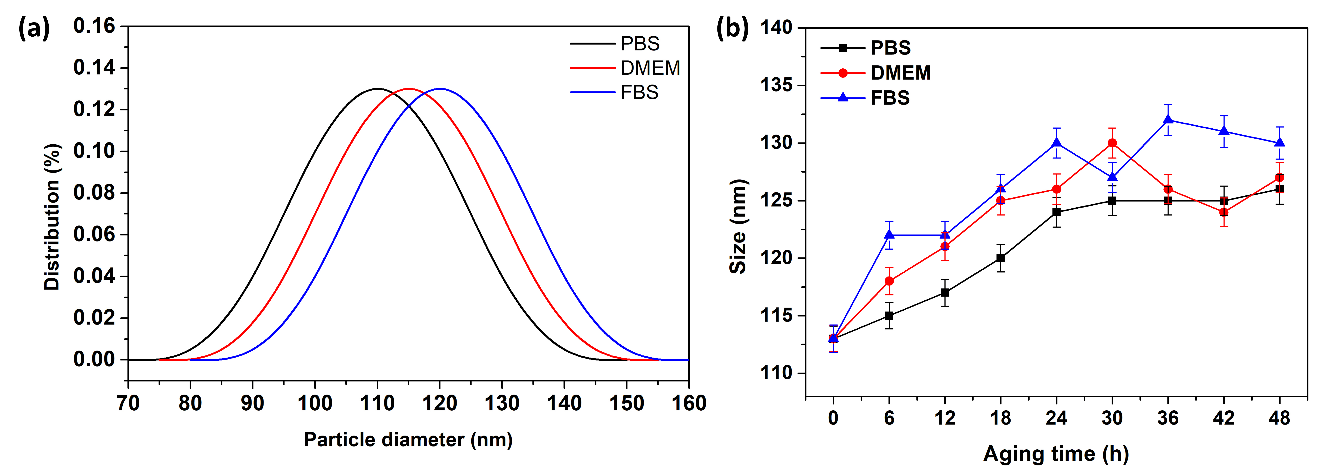


**Fig. S1.** Hydrodynamic size distribution of composite MPB nanoparticles: (a) dispersed in PBS, DMEM, and FBS; (b) stability of the composite MPB nanoparticles with time.

**Stability of synthesized nanocomposite**

Stability determination by UV–vis spectra of the MPB nanocomposite solutions after dissolving in different solutions such as DMEM media, and FBS.


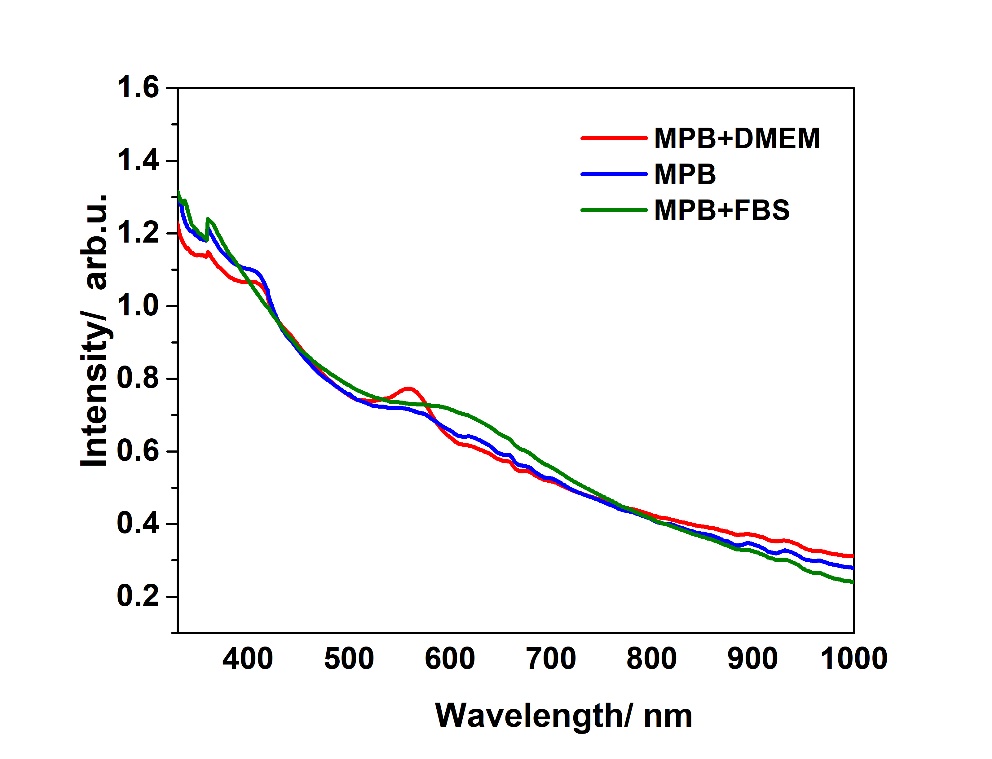


**Fig. S2.** Stability determination by UV–vis–NIR spectra of the MPB nanocomposite.


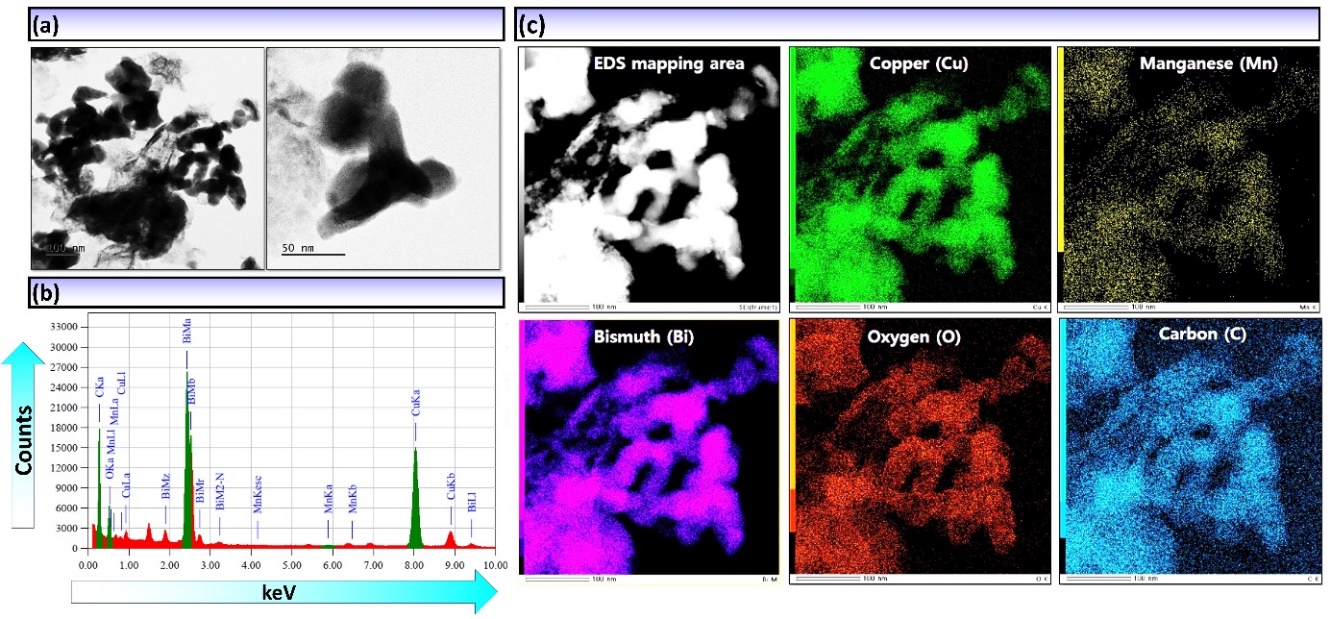


**Fig. S3.** Post photothermal (a) FE-TEM image of MPB nanocomposite. (b) A typical EDS spectrum of MPB nanocomposite. (c) EDS elemental mapping of the nanocomposite.

**Photothermal conversion efficiency of MPB nanocomposites**

To evaluate the photothermal conversion efficiency (*η*), the aqueous solution of **MPB** nanocomposite (250 mg∙mL^-1^) was exposed to the 808 nm laser source at 1.0 W∙cm^-2^. The efficiency was calculated using the relation:

$\eta=\frac{hS (T_{\max}-T_{\mathrm{env}}{) -Q_{\mathrm{dis}}}}{I(1-10{}^{-A808})}$ (i),

where *h* represents the heat transfer coefficient, *S* is the surface area of the container. The maximum steady-state temperature (*T*_max_) of the **MPB** solution was 66.11 °C and the environment temperature (*T*_env_) was 26.47 °C. The temperature change (*T*_max_ − *T*_env_) of the **MPB** nanoparticle solution was 39.64 °C. The laser power density, or irradiance *I,* was 1.0 W∙cm^-2^. A_808_ is the absorbance of the **MPB** nanocomposite at 808 nm. *Q*_dis_ expresses heat dissipated from the light absorbed by the solvent and container.


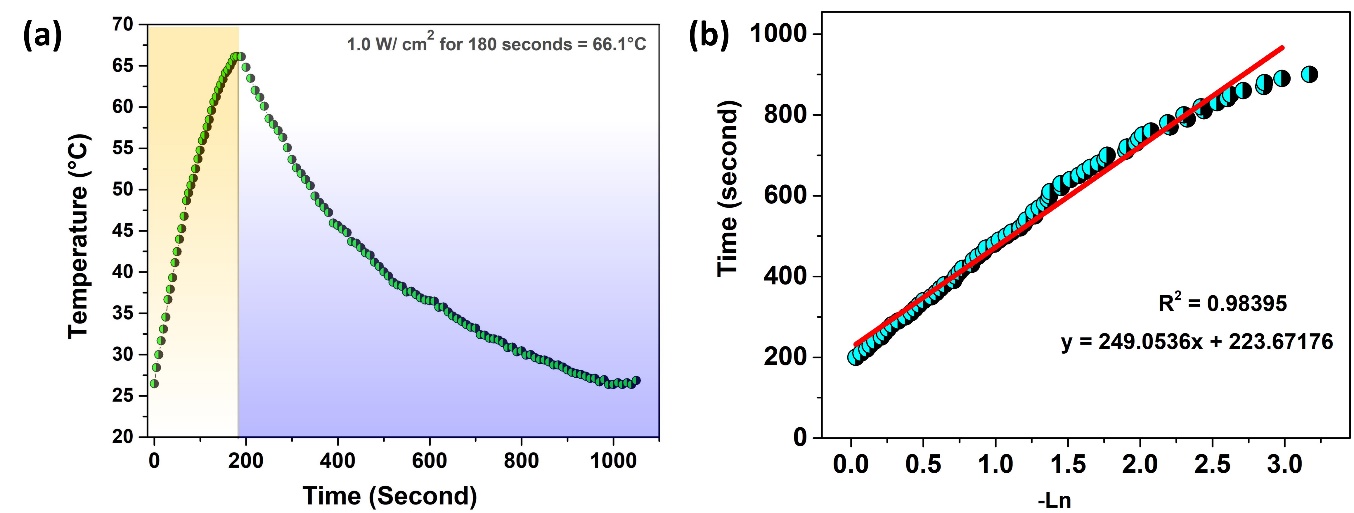


**Fig. S4. (a)** Photothermal effect of the aqueous solution containing Anti-EGFR conjugated MPB nanocomposites with the NIR laser (808 nm, 1.0 W∙cm−2, 180 s), **(b)** Time constant curve fitting for heat transfer of MPB nanocomposites (250 μg∙mL−1) (τs =249.06 s) by applying linear time data vs -Ln(θ) from the cooling stage.

In order to determine *hS*, a dimensionless parameter *θ* was introduced using the maximum system temperature, *T*_max_, correlated by:


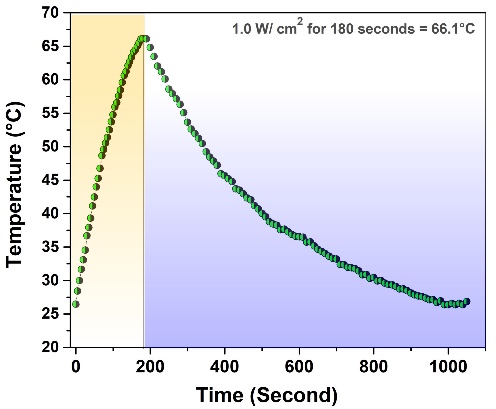


$\theta=\frac{T-T_{\mathrm{env}}}{T_{\max}-T_{\mathrm{env}}}$ (ii)

and a sample system time constant $\tau_{s}$ was calculated using the relation:

${t=-\tau}_{s}\ln\left( \theta\right) (\mathrm{iii})$

where $\tau_{s}$ could be calculated from the previous log figure and is expressed as:

$\tau_{s}=\frac{\sum_{i} m_{i}c_{p,i}}{hS} \left( \mathrm{iv} \right),$

where, $m_{i}c_{p,i}$ is the product of mass (0.2 g) and heat capacity (4.2 J/g∙°C) of water. Applying the linear time data (see the cooling curve in Fig. S3) during the cooling period (after 180 s) vs negative natural logarithm of driving force, $\tau_{s}$ was calculated as 245s, (from log value). The calculated *hS* value was 2.52 mW/ °C. The photothermal conversion efficiency was calculated to be ***η ≈* 33.824%** which is in the acceptable range for efficient biomedical application.

**Table S2.** Photothermal conversion efficiency (*η*) of several PTT agents at 808 nm.

| Sample | *η* [%] | Ref*.* |
| --- | --- | --- |
| Cu_9_S_5_ | 25.7 | [2] |
| Bi_2_S_3_ nanorods | 28.1 | [3] |
| FePt | 30 | [4] |
| PVA-SNT (Ag) | 30.44 | [5] |
| Au nano-shells | 32.63 | [6] |
| Ag@PDA | 41.9 | [7] |
| Anti-EGFR conjugated MPB nanocomposites | **33.82** | Present study |

**Tumor size and volume estimation**

The tumor size and mice weight were measured by digital slide calipers and weight balance. The tumor volumes (*V*) were calculated by the following equation:

$$V=\frac{\pi\times L W^{2}}{6}$$

Here, *V* denotes volume, *L* stands for length, and *W* represents the width of the tumor.

###

**Oxygen generation study**

An experimental study was conducted to assess the production of oxygen using 1,3-Diphenylisobenzofuran (DPBF) as a probe. The study involved examining the dissolved oxygen concentrations in the MPB nanocomposites under various conditions. UV-vis absorption spectra of DPBF mixed under different treatments were recorded to evaluate the generation of singlet oxygen (^1^O_2_) species. The UV absorbance of DPBF mixed with MB solution was utilized as the standard for calculating the efficiency of ^1^O_2_ generation, as depicted in Figure S5.


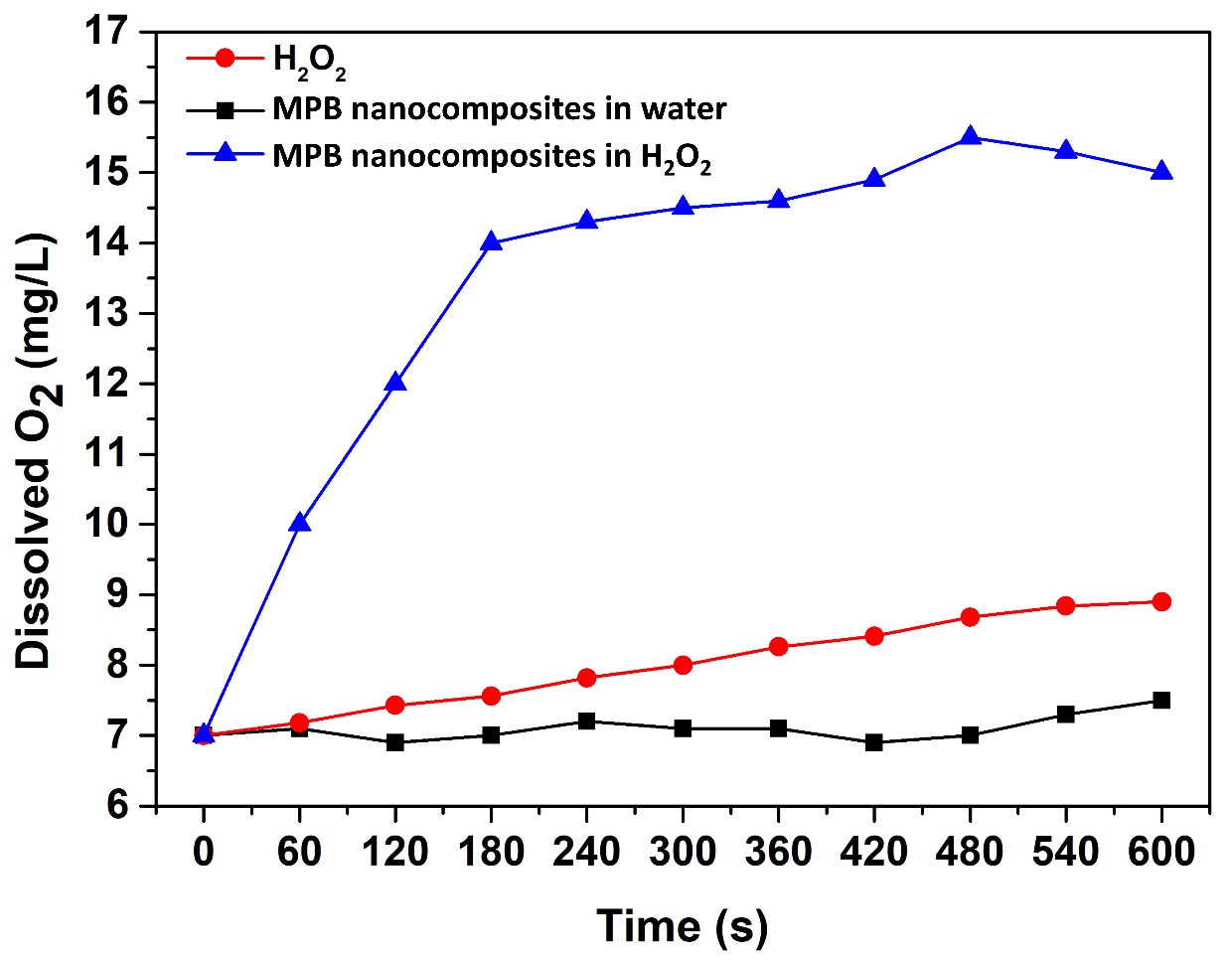


**Fig. S5.** The levels of dissolved oxygen in the MPB nanocomposites were measured under various conditions. UV-vis absorption spectra of DPBF were recorded after mixing it with different treatments for varying durations of irradiation.

**Cell uptake study of nanocomposite**

Following the incubation of Anti-EGFR-conjugated MPB nanocomposites with MDA-MB-231 breast cancer cell lines, B-TEM analysis was conducted. The results demonstrate the internalization of nanocomposites by the MDA-MB-231 cell lines (Fig. S4a). Subsequently, post-PTT B-TEM analysis (Fig. S4b) reveals cell membrane rupture and fragmentation, leading to cell death. This observation is further substantiated by various fluorescence assays. In vitro cellular uptake studies were analyzed using ICP-MS (Supporting Information Fig. S6c). After incubating the cells with the MPB nanocomposite, cells treated for varying times showed increased concentrations of Mn and Bi. This increase is attributed to the internalization or cellular uptake of the MPB molecules. The results indicate that the concentration of Mn is higher than that of Bi. At the 0-hour time point, some residual Bi and Mn were observed, which may be due to surface accumulation of the nanocomposites on the MDA-MB-231 cell lines (Fig. S6c).


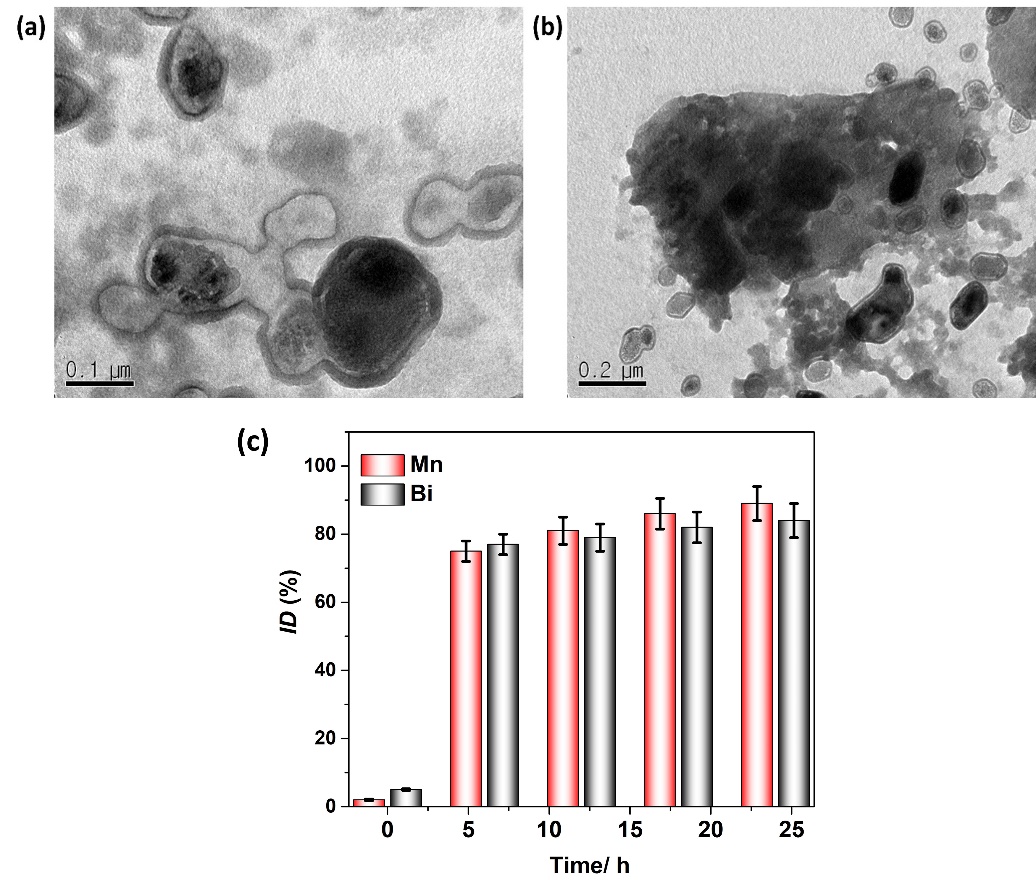


**Fig. S6.** Biological TEM analysis of Anti-EGFR conjugated MPB nanocomposites internalized (a) before photothermal therapy (b) post photothermal therapy in MDA-MB-231 breast cancer cell lines. (c) ICP-MS study of Mn and Bi post 24 h incubation with MBP nanocomposites

### In vitro fluorescence imaging

Various concentrations of anti-EGFR-MPB nanocomposites were incubated with MDA-MB-231 cell lines in a 96-well plate. All samples were analyzed using the OHLABS LUX 4.0 fluorescence imaging system.^7^ The control group consisted of cells without any nanocomposites. As the nanocomposite concentration increased, there was a corresponding increase in fluorescence intensity, as illustrated in Figure S7.


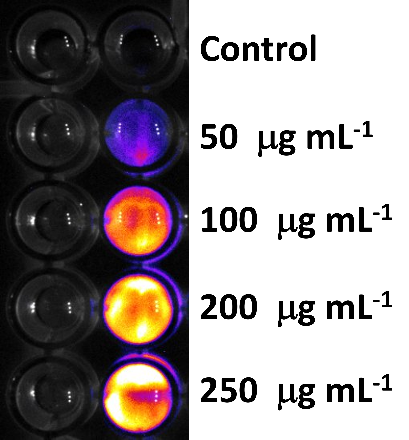


**Fig. S7.** In vitro fluorescence imaging of control cells and MDA-MB-231 cell treated with different concentrations of (50, 100, 200, and 250  μg mL^-1^) anti-EGFR-MPB nanocomposites.

**Estimation of MPB conjugation to the antibody:**

The number of MPB particles conjugated to the antibody was determined using fluorescence spectroscopy. Specifically, labeling MPBs with a fluorescent tag and measuring fluorescence intensity per antibody molecule using a pre-calibrated curve.


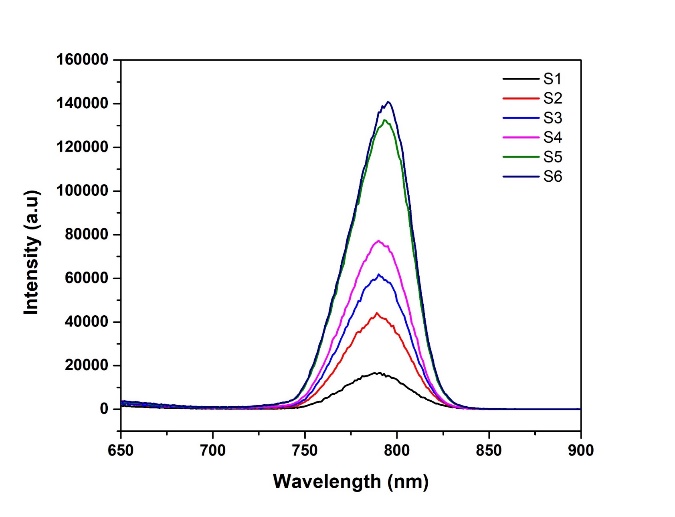


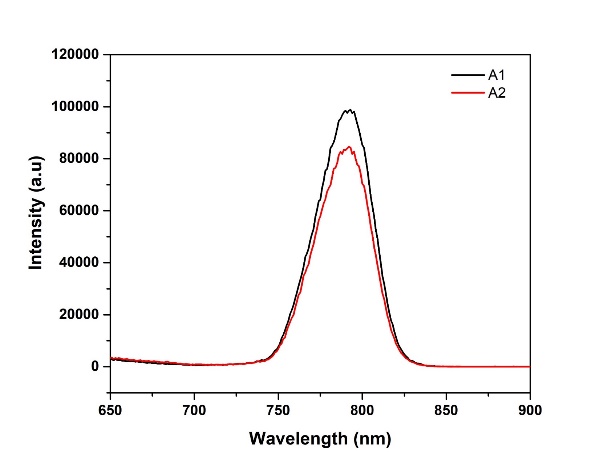
Fig. S8. Fluorescence emission curves for anti-EGFR conjugated MPB particles (S1-S6 correspond to 25-150 micrograms/ mL concentration).

Fig. S9. Result of fluorescence spectroscopy (A1 is the free fluorescence-labeled antibody, whereas A2 is the fluorescence-labeled anti-EGFR-MPB)


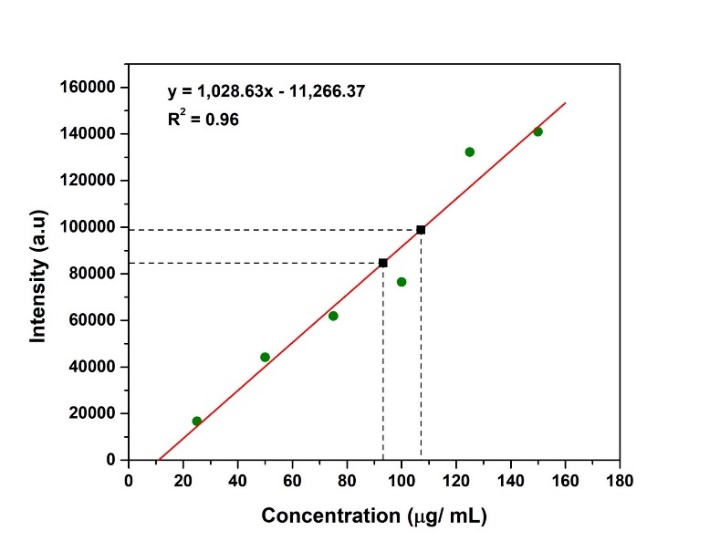


Fig. S10. Calibration curve used to estimate the conjugation efficiency of anti-EGFR with MPB particles.

The concentration of A1 is 107.08 (mg/ mL); A2 is 93.19 (mg/ mL). The estimated antibody conjugation efficiency is ~87.02%.

**References**

[1] Doan VHM, Nguyen VT, Mondal S, Vo TMT, Ly CD, Vu DD, et al. Fluorescence/photoacoustic imaging-guided nanomaterials for highly efficient cancer theragnostic agent. Scientific Reports. 2021;11(1):1-18.

[2] Tian Q, Jiang F, Zou R, Liu Q, Chen Z, Zhu M, et al. Hydrophilic Cu9S5 nanocrystals: a photothermal agent with a 25.7% heat conversion efficiency for photothermal ablation of cancer cells in vivo. ACS Nano. 2011;5(12):9761-71.

[3] Yu M, Zheng J. Clearance pathways and tumor targeting of imaging nanoparticles. ACS Nano. 2015;9(7):6655-74.

[4] Chen C-L, Kuo L-R, Lee S-Y, Hwu Y-K, Chou S-W, Chen C-C, et al. Photothermal cancer therapy via femtosecond-laser-excited FePt nanoparticles. Biomaterials. 2013;34(4):1128-34.

[5] Mondal S, Montaño-Priede JL, Nguyen VT, Park S, Choi J, Doan VHM, et al. Computational analysis of drug free silver triangular nanoprism theranostic probe plasmonic behavior for in-situ tumor imaging and photothermal therapy. Journal of Advanced Research. 2022;41:23-38.

[6] Manivasagan P, Jun SW, Hoang G, Mondal S, Kim H, Doan VHM, et al. Anti-EGFR antibody conjugated thiol chitosan-layered gold nanoshells for dual-modal imaging-guided cancer combination therapy. Journal of Controlled Release. 2019;311:26-42.

[7] Zeng X, Yan S, Di C, Lei M, Chen P, Du W, et al. “All-in-one” silver nanoprism platform for targeted tumor theranostics. ACS Applied Materials & Interfaces. 2020;12(10):11329-40.
