# Supplementary figures and images for: Precision Cancer Therapy Enabled Anti-Epidermal Growth Factor Receptor-Conjugated Manganese Core Phthalocyanine Bismuth Nanocomposite for Dual Imaging-Guided Breast Cancer Treatment
Source: Biomater Res. 2024 Nov 7;28:0092. doi: 10.34133/bmr.0092 (PMC11542904; doi:10.34133/bmr.0092)

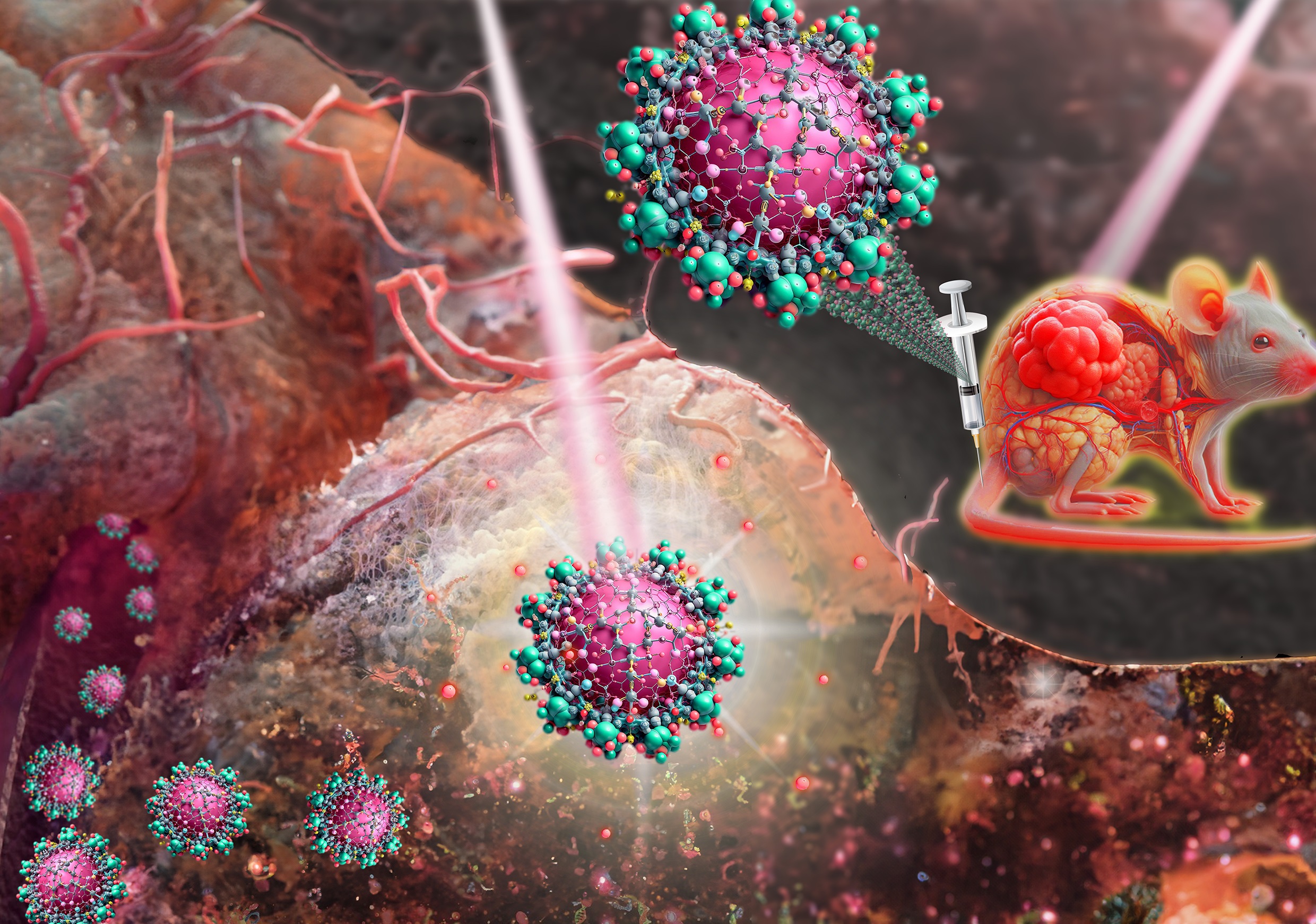

Supplement: Supplementary 1 — Supplementary Text Figs. S1 to S10 Tables S1 and S2 Movies S1 and S2 References [file bmr.0092.f1.zip › Graphical Abstract.jpg]
